# Transcriptional analysis of *Clostridium beijerinckii* NCIMB 8052 to elucidate role of furfural stress during acetone butanol ethanol fermentation

**DOI:** 10.1186/1754-6834-6-66

**Published:** 2013-05-04

**Authors:** Yan Zhang, Thaddeus Chukwuemeka Ezeji

**Affiliations:** 1 The Ohio State University, Department of Animal Sciences and Ohio Agricultural Research and Development Center (OARDC), 305 Gerlaugh Hall, 1680 Madison Avenue, Wooster, OH 44691, USA

**Keywords:** Furfural toxicity, Furfural tolerance, Acetone, Microarray, Real time PCR

## Abstract

**Background:**

Furfural is the prevalent microbial inhibitor generated during pretreatment and hydrolysis of lignocellulose biomass to monomeric sugars, but the response of acetone butanol ethanol (ABE) producing *Clostridium beijerinckii* NCIMB 8052 to this compound at the molecular level is unknown. To discern the effect of furfural on *C. beijerinckii* and to gain insight into molecular mechanisms of action and detoxification, physiological changes of furfural-stressed cultures during acetone butanol ethanol (ABE) fermentation were studied, and differentially expressed genes were profiled by genome-wide transcriptional analysis.

**Results:**

A total of 5,003 *C. beijerinckii* NCIMB 8052 genes capturing about 99.7% of the genome were examined. About 111 genes were differentially expressed (up- or down-regulated) by *C. beijerinckii* when it was challenged with furfural at acidogenic growth phase compared with 721 genes that were differentially expressed (up- or down-regulated) when *C. beijerinckii* was challenged with furfural at solventogenic growth phase. The differentially expressed genes include genes related to redox and cofactors, membrane transporters, carbohydrate, amino sugar and nucleotide sugar metabolisms, heat shock proteins, DNA repair, and two-component signal transduction system. While *C. beijerinckii* exposed to furfural stress during the acidogenic growth phase produced 13% more ABE than the unstressed control, ABE production by *C. beijerinckii* ceased following exposure to furfural stress during the solventogenic growth phase.

**Conclusion:**

Genome-wide transcriptional response of *C. beijerinckii* to furfural stress was investigated for the first time using microarray analysis. Stresses emanating from ABE accumulation in the fermentation medium; redox balance perturbations; and repression of genes that code for the phosphotransferase system, cell motility and flagellar proteins (and combinations thereof) may have caused the premature termination of *C. beijerinckii* 8052 growth and ABE production following furfural challenge at the solventogenic phase.This study provides insights into basis for metabolic engineering of *C. beijerinckii* NCIMB 8052 for enhanced tolerance of lignocellulose-derived microbial inhibitory compounds, thereby improving bioconversion of lignocellulose biomass hydrolysates to biofuels and chemicals. Indeed, two enzymes encoded by Cbei_3974 and Cbei_3904 belonging to aldo/keto reductase (AKR) and short-chain dehydrogenase/reductase (SDR) families have been identified to be involved in furfural detoxification and tolerance.

## Introduction

*Clostridium* species are gram positive, anaerobic, spore-forming bacteria [1]. Production of ABE (acetone-butanol-ethanol) by solventogenic *Clostridium* species is attractive by virtue of their ability to utilize sugars such as cellobiose, glucose, xylose, arabinose and mannose that are present in lignocellulosic biomass hydrolysate. Butanol production from lignocellulosic biomass has shown promise, and the feedstock is abundant, renewable and relatively cheap [2]. However, microbial inhibitors, such as furfural, hydroxymethylfurfural (HMF), hydroxybenzaldehyde, and coumaric acid, are produced during lignocellulosic biomass pretreatment and these compounds impede biofuel production from generated hydrolysates [2].

These lignocellulose-derived inhibitory compounds inhibit microbial cell growth and biofuel production by disrupting cell membranes, damaging polynucleotides, repressing central metabolic enzymes, decreasing intracellular pH, increasing cell turgor pressure, and inducing oxidative stress [3,4]. Although physical, chemical and biological inhibitor removal methods may facilitate substrate utilization and butanol fermentation, removal of inhibitors from hydrolysates prior to fermentation may not be economically feasible due to the cost associated with additional processing steps and the potential loss of fermentable sugars [5]. To make bioconversion of lignocellulosic biomass to butanol economically feasible, development of an inhibitor-tolerant strain of bacteria is crucial. Previous studies have investigated the impact of lignocellulose-derived inhibitors on butanol fermentation. Ezeji et al. [2] have demonstrated the inhibitory effect of corn-fiber-derived aldehyde, organic acid and phenolic compounds on cell growth and ABE production by *C. beijerinckii* BA101, and they have shown the synergistic effect of mixtures of inhibitors over the sum of individual toxic effects. Chemicals present in wheat straw hydrolysates, mainly furfural and HMF, have also been reported to enhance butanol productivity when using *C. beijerinckii* P260 [6]. Furthermore, the detoxification of furfural and HMF by *C. acetobutylicum* ATCC 824 has been shown during butanol fermentation [7]. However, the underlying mechanisms for inhibitor detoxification and tolerance by fermenting solventogenic *Clostridium* species remain unclear. This gap in knowledge continues to hamper attempts at engineering inhibitor-tolerant bacterial strains, as evidenced by the persistent low butanol productivity from biomass feedstock and, hence, the high cost of butanol production.

The objective of this study was to examine the response of *C. beijerinckii* NCIMB 8052 at the mRNA level to the challenge of furfural to better understand the interplay of furfural toxicity and corresponding bacterial tolerance mechanisms. The impact of furfural, the most representative lignocellulose-derived inhibitor, on *C. beijerinckii* NCIMB 8052 was studied to understand its potential mechanism and that of other lignocellulose-derived aldehydes. To gain insight into mechanisms of furfural toxicity and tolerance, the interactive effect of furfural conversion and ABE production was studied by challenging fermentation cultures with different doses of furfural at different growth stages. To examine physiological alterations in *C. beijerinckii* NCIMB 8052 cell growth and ABE production as a consequence of furfural stress, gene expression patterns between control and furfural-stressed treatment cultures were compared by genome-wide transcriptional microarray analysis. The comparison of gene expression patterns in relation to ABE production is expected to provide insights toward metabolically engineering *C. beijerinckii* NCIMB 8052 with enhanced tolerance for lignocellulose-derived microbial inhibitory compounds and better utilization of lignocellulosic biomass hydrolysates.

## Results

### Why microarray analysis of *C. beijerinckii* 8052 transcriptome under furfural stress?

Furfural was chosen as archetypical lignocellulose-derived inhibitory compound for this investigation because it is the most prevalent microbial inhibitor generated during pretreatment and hydrolysis of lignocellulosic biomass to monomeric sugars. However, furfural has been shown previously to enhance solventogenic *C. beijerinckii* BA101 growth and ABE production when the fermentation medium was supplemented with <3 g/L furfural prior to fermentation [2,8]. To better understand mechanisms with which furfural affect *C. beijerinckii* 8052 physiology, the global response of *C. beijerinckii* 8052 to the challenge of furfural during both acidogenesis and solventogenesis at the mRNA level was profiled using whole genome microarray analysis. The findings from the present study are grouped into different attributes.

### Expression of *C. beijerinckii* 8052 redox and cofactor genes in the presence of furfural

**Figure 1:**
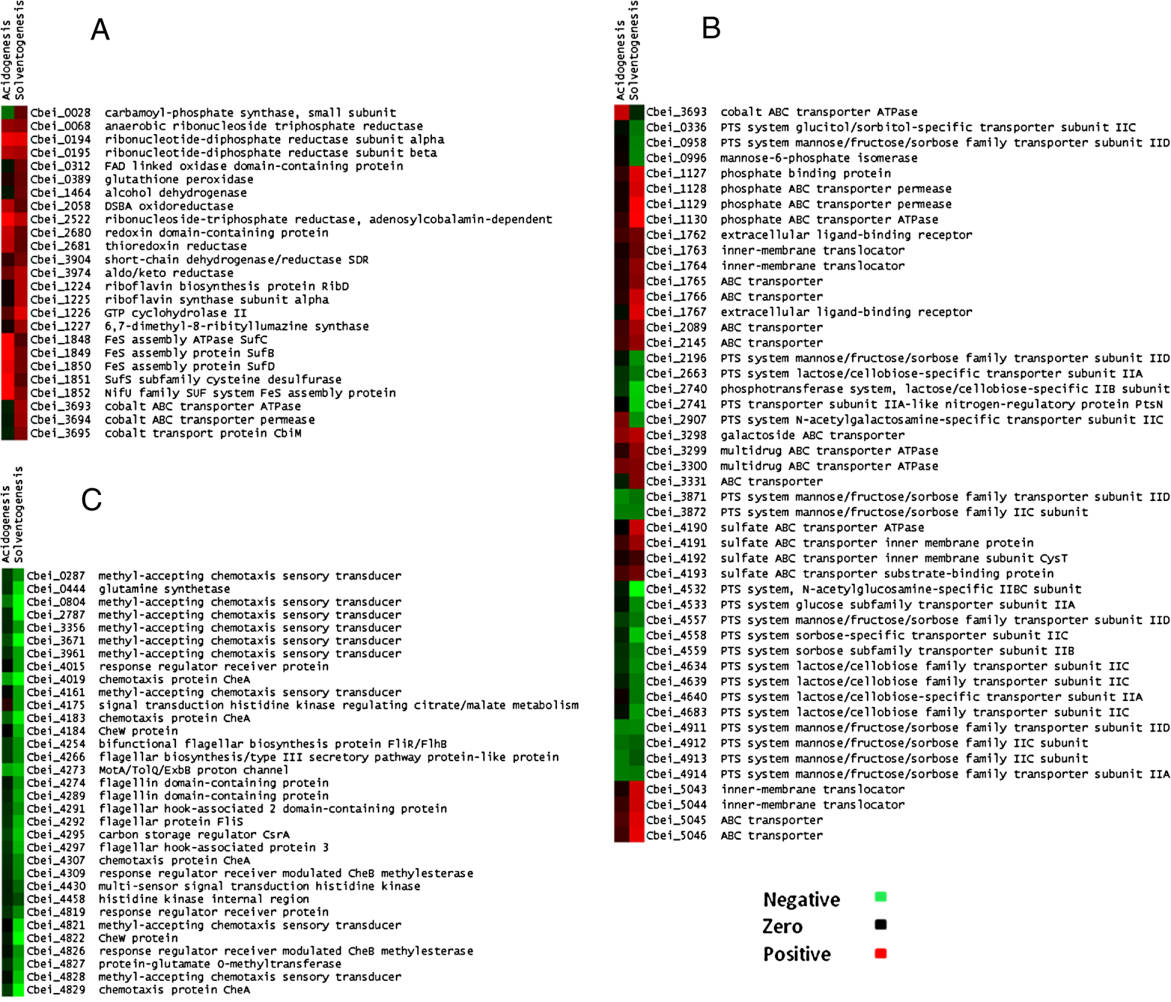
**Comparison of gene expression after furfural challenge at acidogenic and solventogenic phases.** Results are grouped into different attributes: redox and cofactor genes (**A**), membrane transporter genes (**B**), and two-component signal transduction system and chemotaxis genes (**C**).

After the challenge of furfural during the acidogenic phase some genes expressing redox proteins in *C. beijerinckii* 8052 increased by up to 16-fold compared with that in the control group (Figure 1A and Additional file 1: Table S4A). Gene ontology (GO) analysis (Additional file 2: Table S2A) shows that three of these redox proteins are involved in antioxidant activity (GO:0016209): thioredoxin reductase (Cbei_2681), redoxin domain-containing protein (Cbei_2680), and glutathione peroxidase (Cbei_0389); the latter two possess oxidoreductase activity acting on peroxide as an acceptor (GO:0016684) and in response to oxidative stress (GO:0006979). Another group of genes that was up-regulated by more than threefold encodes oxidoreductases acting on CH or CH2 groups, with disulfide as an acceptor (GO:0016728) (Additional file 2: Table S2A). According to KEGG enrichment pathway analysis, this group of genes is associated with purine (cbe00230) and pyrimidine (cbe00240) metabolisms (Additional file 3: Table S3), and includes anaerobic ribonucleoside triphosphate reductase (Cbei_0068), adenosylcobalamin-dependent ribonucleoside-triphosphate reductase (Cbei_2522), and ribonucleotide-diphosphate reductase subunits (Cbei_0194 and Cbei_0195). The remaining oxidoreductases (GO:0016491) (Additional file 2: Table S2A) that had higher expression in the furfural treatment culture than in the control culture are aldo/keto reductase (Cbei_3974), short-chain dehydrogenase/reductase (SDR) (Cbei_3904), DSBA oxidoreductase (Cbei_2058), FAD linked oxidase domain-containing protein (Cbei_0312), and alcohol dehydrogenase (Cbei_1464) (Figure 1A and Additional file 1: Table S4A). The transcriptome of *C. beijerinckii* 8052 after furfural challenge at the solventogenic phase shows some similarities in terms of redox enzymes. All the above genes, except FAD linked oxidase domain-containing protein (Cbei_0312), and alcohol dehydrogenase (Cbei_1464), were also induced by furfural challenge at the solventogenic phase (Figure 1A and Additional file 1: Table S4C).

Besides redox enzymes, components associated with redox reactions were also highly expressed in cultures challenged with furfural at the acidogenic phase. One of the related components affected by furfural treatment is the iron-sulfur cluster. The expression of genes encoding iron-sulfur cluster assembly proteins (Cbei_1848, Cbei_1849, Cbei_1850, Cbei_1851 and Cbei_1852) increased by up to fivefold (Figure 1A and Additional file 1: Table S4A); these genes are classified into the cofactor biosynthetic process (GO:0051188) (Additional file 2: Table S2A). Another group of genes classified into the same group (GO:0051188), as well as into the vitamin biosynthetic process (GO:0009110) (Additional file 2: Table S2A), includes those encoding cobalt ABC transporter ATPase (Cbei_3693), cobalt ABC transporter permease (Cbei_3694) and cobalt transport protein CbiM (Cbei_3695) (Figure 1A and Additional file 1: Table S4A). In addition, differential expression was also observed in furfural-challenged cultures for several members of riboflavin biosynthesis genes (Cbei_1224, Cbei_1225, Cbei_1226, Cbei_1227) (Figure 1A and Additional file 1: Table S4A). This group of genes belongs to the Gene Ontology term riboflavin metabolic process (GO:0006771) (Additional file 2: Table S2A), and if classified by KEGG pathway analysis, these genes are involved in riboflavin metabolism (cbe00740) (Additional file 3: Table S3). However, furfural challenge during solventogenesis affected gene expression differently from that at acidogenesis in terms of redox enzyme cofactors. First, expression of genes that code for iron-sulfur cluster assembly proteins was even higher during solventogenesis (Figure 1A and Additional file 1: Table S4C), and those genes (Cbei_1848, Cbei_1849, Cbei_1850, Cbei_1851 and Cbei_1852) were up-regulated in furfural-challenged cultures by up to 54-fold compared to no more than fivefold during acidogenesis (Figure 1A and Additional file 1: Table S4C). On the other hand, the expression of genes involved in synthesis of other cofactors, including riboflavin and cobalamin, did not show obvious alterations during furfural challenge at solventogenesis (Figure 1A), although these genes were highly induced during furfural challenge at acidogenesis (Figure 1A and Additional file 1: Table S4A).

### Expression of membrane transporter genes in *C. beijerinckii* 8052

Gene expression analysis of *C. beijerinckii* 8052 responding to furfural stress during butanol fermentation was performed to determine not only the effect of furfural on *C. beijerinckii* 8052 growth and ABE production but also on molecular physiological changes. Furfural in the ABE fermentation medium altered expressions of the membrane transport system, including ATP-binding cassette transporters (ABC-transporter) and phosphotransferase system (PTS), in *C. beijerinckii* 8052 during both acidogenic and solventogenic phases (Figure 1B). While some ABC-transporter genes such as galactoside ABC transporter (Cbei_3298), multidrug ABC transporter ATPase (Cbei_3299 and Cbei_3300), and cobalt ABC transporter ATPase (Cbei_3693) were expressed up to sevenfold in furfural-challenged *C. beijerinckii* 8052 at the acidogenic phase (Figure 1B and Additional file 1: Table S4A), expression of these transporter genes was increased by a greater fold during the solventogenic phase (Figure 1B and Additional file 1: Table S4C). According to KEGG pathway analysis, some ABC-transporter-related genes may be classified into what is known as KEGG pathway ABC transporters (cbe02010) (Additional file 3: Table S3), which include transport proteins that catalyze transmembrane movement of different substrates including sulfate, phosphate and branched-chain amino acid. Expression of these genes increased up to twelvefold in furfural-challenged *C. beijerinckii* 8052 during solventogenesis (Figure 1B and Additional file 1: Table S4C). Specifically, genes involved in sulfate transportation include sulfate ABC transporter ATPase (Cbei_4190), sulfate ABC transporter inner membrane protein (Cbei_4191 and Cbei_4192), and sulfate ABC transporter substrate-binding protein (Cbei_4193); phosphate transporters include phosphate binding protein (Cbei_1127), phosphate ABC transporter permease (Cbei_1128 and Cbei_1129), and phosphate ABC transporter ATPase (Cbei_1130); and genes that code for branched-chain amino acids include extracellular ligand-binding receptor (Cbei_1762, Cbei_1767 and Cbei_5042), inner-membrane translocator (Cbei_1763, Cbei_1764, Cbei_5043, and Cbei_5044), and ABC transporter (Cbei_1765, Cbei_1766, Cbei_5045, Cbei_5046, and Cbei_2145). In addition, genes for cyanate or nitrite transportation that belong to ABC transporters (Cbei_2089 and Cbei_3331) are equally induced in furfural-challenged *C. beijerinckii* 8052 during solventogenesis (Figure 1B and Additional file 1: Table S4C).

Unlike ABC-transporter genes whose expressions were increased by furfural-challenged *C. beijerinckii* 8052, another member of the membrane transporter system, phosphotransferase system (PTS), was repressed in furfural-challenged cultures of *C. beijerinckii* 8052 at both acidogenic and solventogenic phases (Figure 1B). Prominent among the PTS are the PTS system mannose/fructose/sorbose family transporters involved in fructose and mannose metabolism (cbe00051) and amino sugar and nucleotide sugar metabolism (cbe00520) (Additional file 3: Table S3). While mostly genes encoding PTS system mannose/fructose/sorbose family transporter subunits IIA (Cbei_4914), IIB (Cbei_4913), IIC (Cbei_4912 and Cbei_3872), and IID (Cbei_4911 and Cbei_3871) were repressed by up to fourfold when cultures of *C. beijerinckii* 8052 were challenged with furfural during the acidogenic growth phase (Figure 1B and Additional file 1: Table S4B), a wider spectrum of genes, including PTS system mannose/fructose/sorbose family transporters, was repressed when cultures of *C. beijerinckii* 8052 were challenged with furfural at the solventogenic growth phase (Figure 1B and Additional file 1: Table S4D). The repressed genes associated with sugar metabolism during the solventogenic growth phase include mannose/fructose/sorbose family transporter subunit IID (Cbei_0958, Cbei_2196, Cbei_3871, Cbei_4557, and Cbei_4911), mannose/fructose/sorbose family IIC subunit (Cbei_3872), sorbose-specific transporter subunit IIC (Cbei_4558), sorbose subfamily transporter subunit IIB (Cbei_4559), mannose-6-phosphate isomerase (Cbei_0996), and glucitol/sorbitol-specific transporter subunit IIC (Cbei_0336) (Figure 1B). Besides the listed genes, N-acetylglucosamine-specific IIBC subunit (Cbei_4532) and glucose subfamily transporter subunit IIA (Cbei_4533) are also involved in amino sugar and nucleotide sugar metabolism (cbe00520) (Additional file 3: Table S3). The repression of these genes may affect the transportation and metabolism of sugars such as those in the glucose family (N-acetyl-D-glucosamine, D-glucosamine and glucosides), the lactose and cellobiose families, the mannose family (mannose and galactosamine), and others such as sorbose, sorbitol, glucitol, and L-ascorbate, many of which are monomeric sugars of lignocellulosic biomass. Furfural challenge of *C. beijerinckii* 8052 during the solventogenic phase, in addition, inhibited other specific PTS systems, including lactose/cellobiose-specific subunits (Cbei_2663, Cbei_2740, Cbei_4634, Cbei_4639, Cbei_4640, and Cbei_4683), sorbose-specific subunits (Cbei_2907) and subunit IIA-like nitrogen-regulatory protein PtsN (Cbei_2741) (Figure 1B and Additional file 1: Table S4D).

### Expression of a two-component signal transduction system, chemotaxis, and cell motility genes in *C. beijerinckii* 8052

As with membrane transporter genes, the expression of genes associated with the two-component signal transduction system (cbe02020) was altered in furfural-challenged *C. beijerinckii* 8052 at both acidogenic and solventogenic phases (Additional file 3: Table S3). Following furfural challenge of *C. beijerinckii* 8052 during the acidogenic growth phase, only two genes (Cbei_4019, chemotaxis protein CheA and Cbei_4273, MotA/TolQ/ExbB proton channel) involved in the coding of two-component signal transduction system were repressed by about fourfold (Figure 1C and Additional file 1: Table S4B). When the *C. beijerinckii* 8052 culture was challenged with furfural at the solventogenic growth phase, more than 40 genes were repressed by up to 18-fold (Figure 1C and Additional file 1: Table S4D). Notably, the two major functional categories of genes belonging to the two-component signal transduction system are bacterial chemotaxis (cbe02030) and flagellar assembly (cbe02040) (Additional file 3: Table S3).

Although chemotaxis is the most widely studied two-component sensory system in bacteria, not much has been reported about the system in relation to furfural stress in solventogenic *Clostridium* species. When the *C. beijerinckii* 8052 culture was challenged with furfural at the solventogenic growth phase, many genes associated with the chemotaxis sensory system in *C. beijerinckii* 8052, such as methyl-accepting chemotaxis sensory transducer (Cbei_0287, Cbei_0804, Cbei_2787, Cbei_3356, Cbei_3671, Cbei_3961, Cbei_4161, Cbei_4821, and Cbei_4828) and genes that code for chemotaxis proteins (CheA Cbei_4307, Cbei_4829, and Cbei_4183; CheB Cbei_4309 and Cbei_4826; CheR Cbei_4827; CheW Cbei_4184 and Cbei_4822; CheY Cbei_4819 and Cbei_4015; and MotA Cbei_4273), were differentially repressed (Figure 1C and Additional file 1: Table S4D). Since chemotaxis directs flagellar motion and controls the swimming pattern of the cell [9], genes encoding flagellar assembly proteins were also differentially repressed by furfural. These flagellar proteins include FliS (Cbei_4292), FliR/FlhB (Cbei_4254), FliH (Cbei_4266), MotA (Cbei_4273), FlgL (Cbei_4297), FliC (Cbei_4274 and Cbei_4289), and FliD (Cbei_4291), as shown in Figure 1C and Additional file 1: Table S4D. Additionally, there are two-component signal transduction systems related to genes encoding proteins that partake in many cellular functions such as quorum sensing and flagella assembly (flagellin domain-containing protein Cbei_4274 and Cbei_4289, and MotA/TolQ/ExbB proton channel Cbei_4273), carbon storage regulation (carbon storage regulator CsrA Cbei_4295), nitrogen assimilation (glutamine synthetase Cbei_0444), and cell cycle progression and development (signal transduction histidine kinase regulating citrate/malate metabolism Cbei_4175, multi-sensor signal transduction histidine kinase Cbei_4430, and histidine kinase internal region Cbei_4458) that were differentially repressed when the *C. beijerinckii* 8052 culture was challenged with furfural at the solventogenic growth phase (Figure 1C and Additional file 1: Table S4D).

### Validation of gene expression data from microarray analysis by Q-RT-PCR

**Figure 2:**
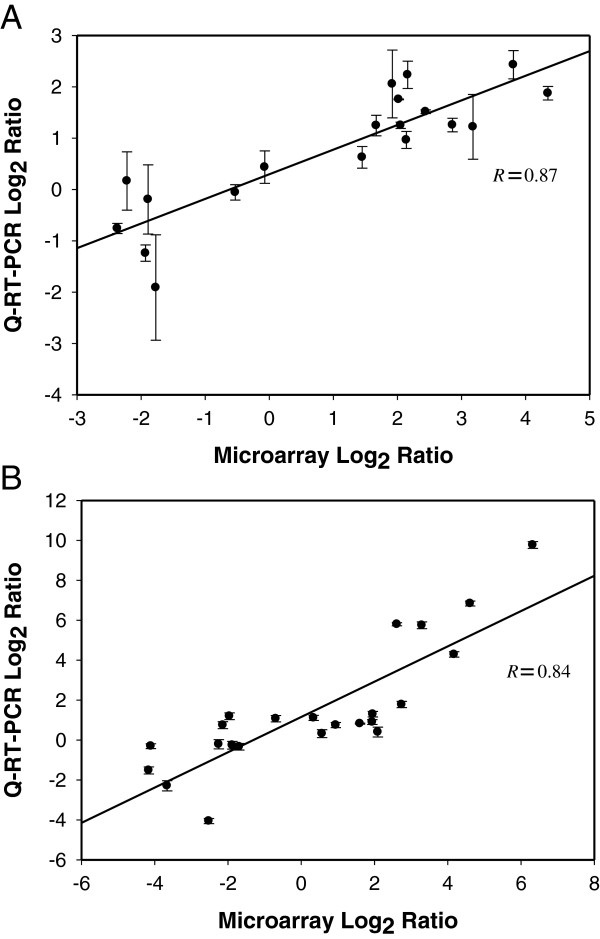
**Validation of Microarray data (gene expression) from *C. beijerinckii *8052 challenged with furfural during two growth phases using Q-RT-PCR.** (**A**) acidogenic phase and (**B**) solventogenic phase.

To validate differential gene expressions obtained using microarray analysis, Q-RT-PCR was applied to quantify gene expression levels in biological replicate cultures of *C. beijerinckii* 8052 using treatment conditions that mimicked microarray treatment but were independent of the cultures used for microarray analysis. Briefly, the *C. beijerinckii* 8052 culture was challenged with furfural at acidogenic and solventogenic growth phases during which 19 and 23 genes, respectively, were evaluated. The genes were selected randomly within each range of fold change. Differential gene expressions in furfural-challenged *C. beijerinckii* 8052 determined via microarray analysis and Q-RT-PCR were found to have a high degree of correlation between them at both acidogenic (R = 0.87) and solventogenic phases (R = 0.84) (Figure 2, Additional file 4: Table S1).

### Interactive effect of furfural reduction and ABE production

**Figure 3:**
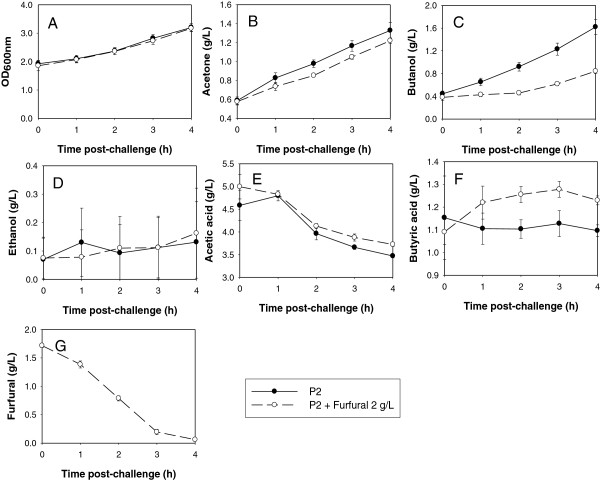
**Cell growth, ABE production and furfural reduction after challenging *C. beijerinckii *8052 with furfural at the acidogenic phase.** Following 8 h of C. beijerinckii 8052 growth in P2 medium, the fermentation broth was distributed into two groups. Each group was made up of 3 aliquots. One group was challenged with 2 g/L furfural (treatment) and the other was left unchallenged (control), followed by anaerobic incubation at 35°C for 4 h during which samples were collected every hour for analysis. Quantified parameters include cell growth (**A**), acetone (**B**), butanol (**C**), ethanol (**D**), acetic acid (**E**), butyric acid (**F**), and residual furfural (**G**).

**Figure 4:**
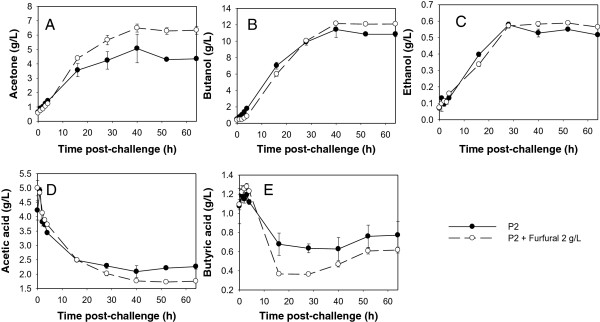
**ABE production by *C. beijerinckii *8052 grown in P2 medium (control) and in P2 medium supplemented with furfural at the acidogenic phase.** Following 8 h of *C. beijerinckii* 8052 growth in P2 medium, the fermentation broth was distributed into two groups. Each group was made up of 3 aliquots. One group was challenged with 2 g/L furfural (treatment) and the other was left unchallenged (control), followed by anaerobic incubation at 35°C for 60 h during which samples were collected every 12 h for analysis. Quantified parameters include acetone (**A**), butanol (**B**), ethanol (**C**), acetic acid (**D**), and butyric acid (**E**).

To determine effects of furfural on *C. beijerinckii* 8052 growth and ABE production at acidogenic and solventogenic phases, a *C. beijerinckii* 8052 culture grown in P2 medium was challenged with furfural, and changes in cell density, acid production and ABE production were measured relative to cultures grown in P2 medium without furfural. Challenge of *C. beijerinckii* 8052 with 2 g/L furfural during the acidogenic phase (fermentation time 8 h) when OD_600_ was between 1.5 and 2.0 resulted in complete depletion of furfural within 4 h, and cell densities of the furfural-challenged *C. beijerinckii* 8052 and control cultures were nearly indistinguishable (Figure 3A). However, acetone and butanol production by furfural-challenged *C. beijerinckii* 8052 only increased by 1.1- and 1.2-fold, respectively, during the period, compared to 1.3- and 2.7-fold increases in acetone and butanol production, respectively, by the control culture (Figure 3A-E). The acetic and butyric acid levels measured in both the furfural-challenged *C. beijerinckii* 8052 and the unchallenged control cultures were reflective of the respective acetone and butanol production profiles (Figure 3). Notably, although ABE production and acid re-assimilation by furfural-challenged *C. beijerinckii* 8052 were inferior to that of the unchallenged control, the fermentation proceeded rapidly following depletion of furfural, and the maximum concentrations of acetone, butanol, and ethanol produced by the furfural-challenged *C. beijerinckii* 8052 were higher than that of the control by 28%, 2% and 6%, respectively (Figure 4A-C). Interestingly, acid assimilation was stimulated in the furfural-challenged *C. beijerinckii* 8052 following furfural depletion in the fermentation medium, and the final concentrations of acetic and butyric acid were lower than that of the control by 22% and 19%, respectively (Figure 4D and E).

**Figure 5:**
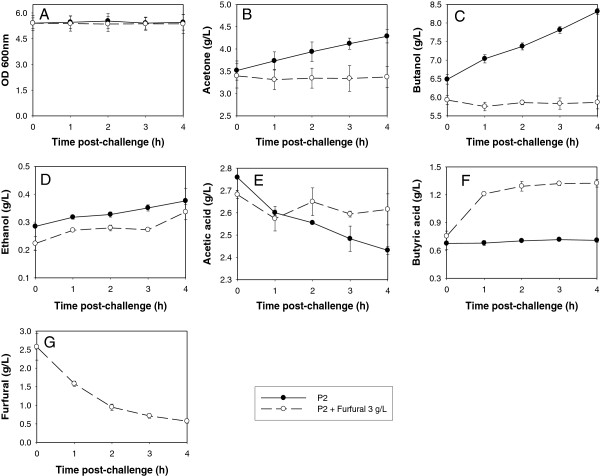
**Cell growth, ABE production and furfural reduction by *C. beijerinckii *8052 culture challenged with furfural (3 g/L) at the solventogenic phase.** Fermentation was monitored for 4 h post-furfural challenge and samples were collected every hour for analyses. Quantified parameters include cell growth (**A**), acetone (**B**), butanol (**C**), ethanol (**D**), acetic acid (**E**), butyric acid (**F**), and residual furfural (**G**).

**Figure 6:**
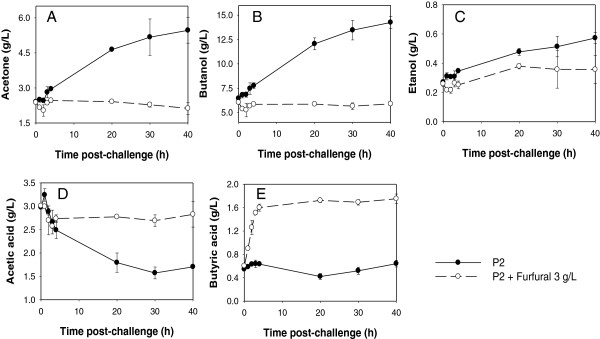
**Cell growth and ABE production by *C. beijerinckii *8052 culture challenged with furfural (3 g/L) at the solventogenic phase.** Fermentation was monitored for 40 h post-furfural challenge. Quantified parameters include acetone (**A**), butanol (**B**), ethanol (**C**), acetic acid (**D**), and butyric acid (**E**).

**Figure 7:**
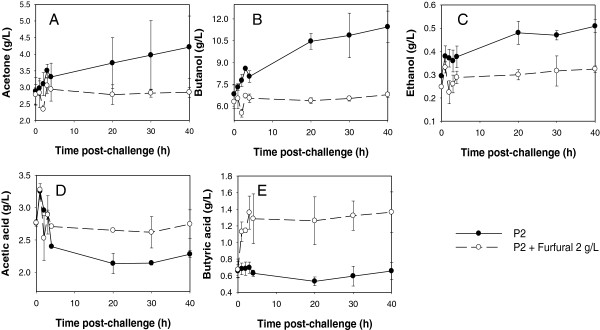
**Acid accumulation and ABE production by *C. beijerinckii *8052 culture challenged with furfural (2 g/L) at the solventogenic phase.** Fermentation was monitored for 4h during which samples were collected every hour for analysis post-furfural challenge. Monitoring was sustained until it was evident that the fermentation has terminated (40 h). Quantified parameters include acetone (**A**), butanol (**B**), ethanol (**C**), acetic acid (**D**), and butyric acid (**E**).

While *C. beijerinckii* 8052 cultures challenged with furfural at the acidogenic phase could tolerate furfural and produce more ABE than the control following the depletion of furfural, challenging *C. beijerinckii* 8052 culture with furfural during the solventogenic phase (fermentation time 25 h; OD_600_ 5.0-5.5) resulted in shut down of ABE production and rapid accumulation of acetic and butyric acid in the fermentation medium (Figure 5). Unlike the control, *C. beijerinckii* 8052 grown in P2 medium without furfural underwent a normal fermentation process (Figures 5 and 6). At solventogenesis, furfural reduction was impeded (Figure 5G) when concentrations of acetone, ethanol and butanol were high (3.40 g/L ± 0.27 g/L, 0.22 g/L ± 0.02 g/L, and 5.93 g/L ± 0.12 g/L, respectively) (Figure 5B and C). Although the cell density of *C. beijerinckii* 8052 in the solventogenic phase culture was four times higher than in the acidogenic phase culture, 3 g/L furfural was reduced by only 80% in 4 h (Figure 5G). To further evaluate the effect of challenging *C. beijerinckii* 8052 at the solventogenic phase with 3 g/L furfural, the fermentation was allowed to proceed for another 40 h during which no further growth and reduction of furfural were observed (data not shown); and uptake of acetic and butyric acid by *C. beijerinckii* 8052 did not occur (Figure 6). Similarly, when 2 g/L furfural was used to challenge *C. beijerinckii* 8052 at the solventogenic phase, the 2 g/L furfural was depleted before 2 h. However, ABE production was shut down (Figure 7A-C) followed by accumulation of acetic and butyric acids (Figure 7D-E) in the fermentation medium, and the culture did not recover following the depletion of furfural.

**Figure 8:**
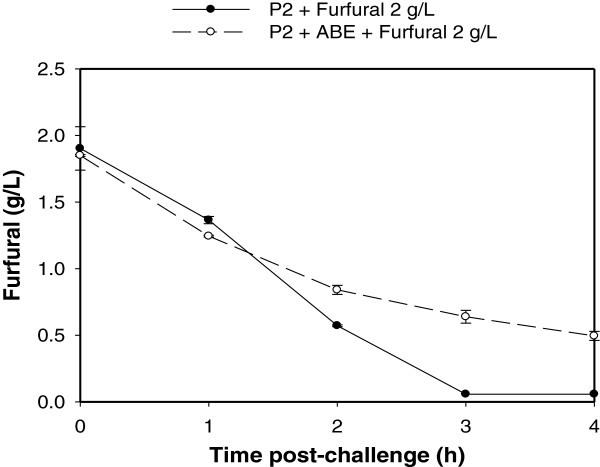
**Reduction of furfural by acidogenic *C. beijerinckii *8052 culture challenged with furfural (2 g/L), acetone (2.80 g/L), ethanol (0.15 g/L) and butanol (5.53 g/L) to bring the final concentrations of acetone, ethanol, and butanol in the fermentation broth to 3.40 g/L, 0.22 g/L, and 5.93 g/L, respectively.**

To independently verify whether the presence of ABE in the fermentation medium was contributing to the toxicity of furfural to *C. beijerinckii* 8052 and decreasing furfural reduction during the solventogenic phase, the acidogenic phase culture of *C. beijerinckii* 8052 was supplemented with 2 g/L furfural together with acetone, ethanol and butanol at concentrations that mimic their concentration at the solventogenic phase. Interestingly, this situation reduced the concentration of furfural in the fermentation medium by only 75% after 4 h post-furfural challenge, unlike the control without ABE supplementation, which depleted the furfural in 3 h (Figure 8). However, the presence of furfural in the fermentation medium during the solventogenic growth phase did not have remarkable impact on the expression of ABE production genes in *C. beijerinckii* 8052 (Additional file 5: Table S5).

## Discussion

While inhibitory properties of degradation products of lignocellulosic biomass are widely recognized as a major limitation to bioconversion of biomass to biofuels and chemicals [5], the gap in knowledge with respect to detoxification of these lignocellulose-derived inhibitory compounds by fermenting microorganisms continues to impede the development of inhibitor-tolerant strains vis–à–vis commercialization of biofuels. This study presents the physiological changes and transcriptional responses of *C. beijerinckii* NCIMB 8052 to furfural challenge at different growth and fermentation stages, highlighting a systematic pattern of gene regulations and revealing potential target genes for strain improvements by genetic engineering.

Genome-wide microarray analysis demonstrated a clear perspective on the effect of the lignocellulosic biomass-derived inhibitor furfural on the transcriptional profile of *C. beijerinckii* 8052. This study revealed for the first time that changes in physiological activities of furfural-challenged cultures of *C. beijerinckii* are coordinated with transcriptional variations during ABE fermentation. Validation of microarray data by Q-RT-PCR using samples from independent biological treatment showed high degrees of correlation coefficient (R) at both acidogenic and solventogenic phases (0.87 and 0.84, respectively), which fall into the upper values of the reported range (−0.48 to +0.93) [10], thus, confirming strong reliability of data obtained by microarray analysis. This result is significant because the correlation coefficient is the generally accepted criterion for assessing reliability of microarray data [11]. Ramifications of obtained results are discussed below under different attributes.

### Redox and cofactor genes are crucial for detoxification of furfural by *C. beijerinckii* 8052

Furfural challenge increases the expression of genes encoding redox proteins in *C. beijerinckii* 8052 (Figure 1A and Additional file 1: Table S4A and S4C). Differential expression of redox genes involved in antioxidant activity suggests that furfural causes oxidative stress in *C. beijerinckii* 8052. In yeast, furfural induces the accumulation of reactive oxygen species (ROS), that are known to damage DNA, lipids and proteins and that subsequently induce programmed cell death [4]. Glutathione peroxidases and thioredoxin peroxidases, which were differentially induced in furfural-challenged *C. beijerinckii* 8052 (Figure 1A and Additional file 1: Table S4A and S4C), can reduce H_2_O_2_ to H_2_O via oxidation of thiol groups. The reduction of oxidized glutathione and thioredoxin is catalyzed by glutathione reductase or thioredoxin reductase, respectively, using NADPH as the electron donor [12]. Thioredoxin and glutathione can also function as oxygen quenchers and hydroxyl radical scavengers [13-15]. Given that this study was conducted in an anaerobic chamber with less than 1 ppm of molecular oxygen (monitored by an oxygen detector), the origin of ROS is not clear. However, production of ROS, hydroperoxide or other radical species by anaerobes has been hypothesized and elucidated previously [16,17]. Organic hydroperoxide generated under anaerobic condition, therefore, may induce the expression of alkyl hydroperoxide reductase gene in *E. coli*[17], and glutathione peroxidase and thioredoxin peroxidase genes in *C. beijerinckii* 8052. Another gene encoding a thioredoxin family protein dsbA oxidoreductase, a periplasmic oxidoreductase that facilitates disulfide bond formation in proteins, was found in this study to be induced by furfural. Overexpression of dsbA in *E. coli* increased soluble protein level in the periplasm and improved enzyme secretion and activity [18]. Elevated levels of antioxidant activity due to furfural challenge indicate increased oxidative stress, thus, accentuating innate detoxification capabilities of *C. beijerinckii* under the influence of furfural stress. Additionally, thioredoxin and thioredoxin reductase work in tandem with ribonucleotide reductase during reduction of ribonucleoside diphosphates to deoxyribonucleoside diphosphates [19]. The induced expression of these redox enzymes (Figure 1A and Additional file 1: Table S4A) involved in purine and pyrimidine metabolism in *C. beijerinckii* 8052 (Additional file 3: Table S3) suggests greater demand of nucleotides due to furfural stress. This premise is supported by the fact that DNA molecules are prone to damage in the presence of furfural [3], and in this case, DNA repair or biosynthesis is activated leading to induced expression of redox enzymes.

The differentially induced genes encoding oxidoreductases such as aldo/keto reductase (AKR) and short-chain dehydrogenase/reductase (SDR) in *C. beijerinckii*, which are involved in the reduction of furfural to furfuryl alcohol, have been reported elsewhere as scavengers of furfural in *Escherichia coli*[20,21], *Saccharomyces cerevisiae*[22], and *Zymomonas mobilis*[23] fermentations. Direct reduction of furfural to the less toxic furfuryl alcohol [7] is another strategy *C. beijerinckii* 8052 uses to mitigate toxic effects of furfural.

Moreover, differential expression of genes encoding the iron-sulfur cluster and cobalamin- and riboflavin-associated proteins, was observed in furfural-challenged *C. beijerinckii* 8052 (Figure 1A and Additional file 1: Table S4A), thus, accentuating cellular responses to furfural stress by redox balancing because these proteins require cofactors such as NADH and NADPH to facilitate catalysis. Notably, the iron-sulfur cluster plays important roles in electron transfer by redox enzymes, disulfide reduction by ferredoxin:thioredoxin reductase, regulation of gene expressions associated with Ferredoxin-NADP^+^ reductase, and iron and sulfur storage in ferredoxins [24]. Similar to iron-sulfur clusters, cobalamin, known as vitamin B12, can also function as redox enzyme cofactors [25]; a typical example is the cobalamin-mediated biodegradation of chloroform by the methanogenic consortium obtained from an anaerobic distillery waste water treatment plant [26]. Riboflavin, the redox active moiety of flavin adenine dinucleotide (FAD) and flavin mononucleotide (FMN), has been shown to be differentially induced in response to furfural challenge during ethanol production by *S. cerevisiae*[27]. Broadly, these results support the idea that induction of genes encoding redox proteins and cofactors and transformation of furfural to less toxic furfuryl alcohol are important mechanisms that *C. beijerinckii* 8052 uses to restore redox balance under furfural stress and mitigation of toxic effects of furfural on the cell.

### Membrane transporter genes play active role in furfural tolerance and detoxification by *C. beijerinckii* 8052

Fluctuations in differential expressions of the membrane transport system, including ATP-binding cassette transporters (ABC-transporter) and phosphotransferase system (PTS), signify possible physiological adaptations in *C. beijerinckii* 8052 in response to furfural stress (Figure 1B). The increased expression of sulfate ABC transporter genes (Figure 1B) in *C. beijerinckii* 8052 may be interpreted following a previously proposed model in *E. coli*[28] in which furfural depresses sulfur assimilation with concomitant inhibition of cell growth, but supplementing the fermentation medium with sulfur-containing amino acids (cysteine and methionine) reversed cell growth and increased cell tolerance to furfural. It is plausible that under furfural stress, *C. beijerinckii* 8052 may sense sulfur limitation and consequently elevate expression of sulfate ABC transporter genes in preparation for potential increased absorption of sulfur from the fermentation medium. It is important to note that there are sulfates (MgSO_4_, MnSO_4_, and FeSO_4_) in the P2 growth medium.

The expression of the phosphate-specific transport (*Pst*) system in *C. beijerinckii* 8052 was significantly enhanced under furfural stress (Figure 1B and Additional file 1: Table S4A). Phosphate is an essential component of nucleotides; hence, it plays a central role in chemical energy and DNA/RNA synthesis. The elevated expression of the *Pst* system may indicate shortage of intracellular phosphates, thus, the need for increased absorption of phosphorus from the environment. Elsewhere, while *Pst* in *E. coli* was demonstrated to have decreased expression in the presence of excessive inorganic phosphate, phosphate limitation induces the expression of *Pst*[29]. Similarly, elevated expression of multiple operons encoding ABC transporters for branched-chain amino acid transportation was observed (Figure 1B and Additional file 1: Table S4A and S4C). It is conceivable that the biosynthesis of branched-chain amino acids (leucine, isoleucine and valine) in *C. beijerinckii* 8052 is perturbed when furfural is present in the medium, hence, the induction of genes encoding a related membrane transportation system to mitigate the perturbation. This line of reasoning agrees with the fact that furfural induces the accumulation of reactive oxygen species and superoxide anions, which may damage the synthesis of amino acids, especially the branched chain amino acids [30]. Therefore, it is reasonable that *C. beijerinckii* 8052 increases the expression of these ABC transporters to facilitate enhanced absorption of exogenous amino acids.

In contrast to ABC transporters, the phosphotransferase system (PTS) reveals decreased expression in furfural-challenged *C. beijerinckii* 8052 (Figure 1B and Additional file 1: Table S4B and S4D). Since the bacterial PTS plays crucial roles in sugar reception, transport and phosphorylation in addition to regulation of catabolic pathways [31], the expression level of PTS may reflect the physiological state of cell metabolism and, consequently, could rationalize the low ABE production and premature termination of the *C. beijerinckii* 8052 fermentation process after furfural challenge at the solventogenic growth phase (Figures 5, 6, 7).

### Furfural influences the adaptation machinery of *C. beijerinckii* 8052

The two-component signal transduction system (TCS) is a stimulus–response coupling signal transduction machinery that allows bacteria to respond and adapt to changes in a wide range of environmental conditions, such as nutrient assimilation [32], cellular redox state [33] and bacterial virulence regulation [34]. Chemotaxis, controlled by TCS, is the cells’ response to stressful environments. In chemotaxis, signals are first sensed by transmembrane receptors known as methyl-accepting chemotaxis proteins (MCPs), which control the autophosphorylation of a kinase protein and then a regulator protein. The regulator protein interacts directly with flagellar proteins that act as motor switches and, thus, controls the swimming pattern of the bacterial cell [35]. Exposure of *C. beijerinckii* 8052 to furfural stress elicits repression of genes that code for MCPs, CheA, CheY, and flagellar proteins (Figure 1C), plausibly causing temporary (at the acidogenic phase) and permanent (at the solventogenic phase) defects in the adaptation machinery of *C. beijerinckii* 8052.

In bacteria, the carbon storage regulator (CsrA) is recognized as an activator of glycolysis, acetate metabolism, and flagellum biosynthesis [36] and as a global regulator of bacterial virulence and stress response [37]. *E. coli csrA*^−^ (*csrA* deficient) strains are known to have severe growth problems due to central carbon stress [38], and the *csrA*^−^ strain of *Helicobacter pylori* significantly attenuates its virulence [37]. In the presence of furfural, the global regulator *CsrA* in *C. beijerinckii* 8052 was significantly repressed (Figure 1C and Additional file 1: Table S4B and S4D), which may result in the repression of glycolysis and consequently, may trigger repertoires of transformations in stationary-phase physiology [38]. This could rationalize the low ABE production and premature termination of the *C. beijerinckii* 8052 fermentation process following furfural challenge at the solventogenic growth phase (Figures 5, 6, 7).

Furthermore, repression of glutamine synthetase (GS) in *C. beijerinckii* 8052 during ABE fermentation in the presence of furfural may decrease the production of glutamine, which may have undesirable effects with respect to nutrient assimilation and cellular redox balance. The GS strain (glutamine-requiring strain) of *Bacillus subtilis* was found to cause pleiotropic effects on glucose catabolite repression [39]. Moreover, glutamine is a precursor of glutamate, which may be used to synthesize glutathione, an important cellular antioxidant (albeit in reduced form) that mitigates stresses [40]. Since the product of GS plays an important role in cellular redox balance, the repression of GS may impair the tolerance of *C. beijerinckii* 8052 to furfural.

### Basis for both stimulatory and inhibitory effects of furfural on *C. beijerinckii* 8052

Addition of furfural (<3 g/L) to the fermentation medium inhibits ABE production by *C. beijerinckii* 8052 to various degrees regardless of the growth stage (acidogenic or solventogenic) of the culture (Figures 3 and 5). While *C. beijerinckii* 8052 challenged with furfural during the acidogenic phase experienced short-term ABE production inhibition (Figure 3), rapid depletion of furfural in the fermentation medium (Figure 3G), full recovery even with elevated cell growth (data not shown), and increase in ABE production following exhaustion of furfural in the growth medium (Figure 4), *C. beijerinckii* 8052 challenged with furfural at the solventogenic growth phase resulted in immediate termination of ABE fermentation (Figures 5 and 6). This finding partly agrees with previous investigations [2,7], which reported that furfural could stimulate growth and ABE production when added at the beginning of fermentation, but it also expands knowledge in the field by uncovering the fact that furfural is most toxic to *C. beijerinckii* 8052 during the solventogenic growth phase.

*C. beijerinckii* 8052 did not recover from the toxic effect of furfural when it was challenged with it at the solventogenic growth phase, yet why does furfural enhance growth of *C. beijerinckii* 8052 when it is added either at the beginning of fermentation or during the acidogenic growth phase? The answer may be found in the genes. Genes *GrpE*, *DnaK* and *DnaJ* in *DnaK* operon encoding GrpE, DnaK and DnaJ proteins are induced under furfural stress (Additional file 1: Table S4A), and they play an important role in mitigating harmful effects of environmental stresses such as UV irradiation [41], ethanol [42], and butanol [43] on microorganisms, and stresses on the cellular chaperone machinery [44]. While GroES and GroEL in the *groE* operon [which are also highly conserved molecular chaperons and are known to be induced by the presence of butanol [45] were differentially induced by more than threefold when *C. beijerinckii* 8052 was challenged with furfural during the solventogenic growth phase (Additional file 1: Table S4C), the operon was differentially induced by less than threefold when *C. beijerinckii* 8052 was challenged with furfural during the acidogenic growth phase (Additional file 1: Table S4A). Notably, overexpression of groES and groEL increases production of ABE, tolerance to toxic products, and metabolism in solventogenic *Clostridium* species [45], but their increased expression during the solventogenic phase was perhaps to overcome (albeit increased expression was not enough to overcome furfural toxicity) the high toxicity of furfural to *C. beijerinckii* 8052 at this physiological growth phase.

Then, why is furfural more toxic to *C. beijerinckii* 8052 during the solventogenic phase than during the acidogenic phase? Three hypotheses are proposed. First, the presence of ABE enhances cell membrane fluidity and inhibits cell metabolism [46], which leads to significant loss of cell functions and weakening of the cellular defense system to furfural. This was underscored by the fact that *C. beijerinckii* 8052 was unable to completely reduce 2 g/L furfural in the presence of ABE during acidogenic growth phase, unlike the control without ABE, which reduced the entire amount of furfural (Figure 8). Second, the biotransformation of furfural, which is catalyzed by NAD(P)H-dependent oxidoreductase [7], competes with NAD(P)H-dependent dehydrogenase (that catalyzes alcohol production) for NAD(P)H coenzymes [47]. The need for NAD(P)H by NAD(P)H-dependent oxidoreductase to boost cellular defense against furfural is a high priority, which leads to a decrease in the NAD(P)H pool and subsequently impedes alcohol production by NAD(P)H-dependent dehydrogenase. This hypothesis is supported by a previous finding in ethanologenic *E. coli*[20,48], wherein silence of an oxidoreductase involved in furfural conversion relieves the diversion of NAD(P)H away from other important biosynthetic processes, thus, increasing cell growth and furfural tolerance. Competition between oxidoreductase and alcohol dehydrogenase for NAD(P)H is severe at the solventogenic growth phase, during which NAD(P)H is needed for the conversion of butyryl-CoA to butyrylaldehyde and subsequently to butanol, unlike the acidogenic growth phase, during which acids are produced in tandem with NAD(P)H production [46]. Third, while furfural repressed the expression of only two genes involved in cell motility by more than threefold when *C. beijerinckii* 8052 was challenged with furfural during the acidogenic phase, more than forty genes were differentially repressed by up to 18-fold when *C. beijerinckii* 8052 was challenged with furfural at the solventogenic phase (Figure 1C and Additional file 1: Table S4B and S4D). Notably, the non-motile strain of *C. acetobutylicum* has been shown to produce lower ABE than the motile parent strain [49].

## Conclusions

While elevated expression of redox and cofactor genes, heat shock genes, and redox balancing may contribute to enhanced ABE fermentation when *C. beijerinckii* 8052 was challenged with furfural at the acidogenic phase, stresses emanating from ABE production; redox balance perturbations; and repression of genes that code for the phosphotransferase system, cell motility and flagellar proteins (and combinations thereof) may have caused the premature termination of *C. beijerinckii* 8052 growth and ABE production following furfural challenge at the solventogenic phase. Transcriptomic and fermentation studies carried out in this work provided a new multifarious basis for both stimulatory and inhibitory effects of furfural on *C. beijerinckii* 8052 during ABE fermentation. Collectively, this study provided insights that could form the basis for metabolic engineering of *C. beijerinckii* NCIMB 8052 for enhanced tolerance of lignocellulose-derived microbial inhibitory compounds, thereby improving bioconversion of lignocellulosic biomass hydrolysates to biofuels and chemicals. Indeed, we have successfully overexpressed Cbei_3974 and Cbei_3904 genes belonging to aldo/keto reductase (AKR) and short-chain dehydrogenase/reductase (SDR) families in *C. beijerinckii* NCIMB 8052, and generated furfural tolerant strains. Detailed physiological and biochemical characterization of developed strains are currently being conducted in our laboratory.

## Materials and methods

### Bacterial strains, culture conditions

*C. beijerinckii* NCIMB 8052 (ATCC 51743) was obtained from the American Type Culture Collection (Manassas, VA) and was used in all experiments unless noted otherwise. Stocks of *C. beijerinckii* 8052 spores were stored in sterile, double-distilled water at 4°C. To revive *C. beijerinckii* 8052 spores, 200 μL stock was heat-shocked for 10 min at 75°C followed by cooling on ice. The heat-shocked spores were inoculated into 10 mL anoxic pre-sterilized tryptone–glucose–yeast extract (TGY) medium and incubated in an anaerobic chamber (Coy Laboratory Products Inc., Ann Arbor, Michigan) with a modified atmosphere of 82% N_2_, 15% CO_2_, and 3% H_2_ for 12 h to 14 h at 35°C ± 1°C until active growth (OD_600_ 0.9–1.1) was attained [50].Eight milliliters of actively growing culture was subsequently transferred into 92 mL of anoxic TGY medium. The culture was grown anaerobically at 35°C ± 1°C for 4 h to 5 h; during this time it reached an optical density (OD_600_) of 0.9 - 1.1. This was used as the pre-culture.

### Furfural-challenged experiments and ABE fermentation

Batch ABE fermentation by *C. beijerinckii* 8052 was performed in 250-mL Pyrex screw-capped media bottles containing 200 mL anoxic P2 medium (glucose 60 g/L and yeast extract 1 g/L) and P2 stock solutions as described previously [7].

To evaluate the response of *C. beijerinckii* 8052 to furfural during acidogenic and solventogenic phases, a 1-L flask containing 600 mL anoxic P2 medium plus P2 stock solutions was inoculated (6% v/v) with *C. beijerinckii* 8052 pre-culture and incubated anaerobically for 8 h; during this time the OD_600_ attained 1.4 ± 0.05 (acidogenic phase). The culture was then subdivided into aliquots of 100 mL in six 150-mL Pyrex screw-capped media bottles. Three bottles in the treatment group were challenged with furfural (2 g/L) and the other three bottles were left unchallenged as the control. After 2 to 3 h growth at 35°C, the original concentration of furfural in the growth medium was reduced by more than half, and *C. beijerinckii* 8052 samples were collected from each bottle and triplicate control and furfural-challenged samples were pooled separately. Notably, cell density of *C. beijerinckii* 8052 during solventogenic phase was markedly higher than that at acidogenic growth phase. Given the greater number of *C. beijerinckii* 8052 cells in the fermentation medium at solventogenic phase; we decided to increase the concentration of furfural to 3 g/L. For furfural challenge at solventogenic phase, 600 ml of *C. beijerinckii* 8052 culture was incubated anaerobically for 24 h; during this time the OD_600_ attained 4.0 ± 0.2, and then subdivided into aliquots of 100 mL in six 150-mL bottles. Three bottles were challenged with 3 g/L of furfural which were the treatment group and the other three bottles were unchallenged (control). Following 2 to 3 h post-furfural challenge, during which time the original concentration of furfural in the growth medium was reduced by more than half, *C. beijerinckii* 8052 samples were collected from each bottle and triplicate control and furfural-challenged samples were pooled separately for analysis. Aliquots of *C. beijerinckii* 8052 samples were placed on ice immediately after removal from the bottles. *C. beijerinckii* 8052 pellets were obtained by centrifugation at 5000 g for 10 min at 4°C prior to suspension in a solution containing a 2:1 ratio of RNAprotect cell reagent to phosphate-buffered saline (PBS) (Qiagen Inc., Valencia, CA) to stabilize the RNA. The suspension was incubated at room temperature for 5 min, centrifuged to obtain cell pellets, and stored at −80°C overnight as described previously [51].

The pH profile of *C. beijerinckii* 8052 fermentation was monitored with a Beckman Ф500 pH meter (Beckman Coulter Inc., Brea, CA). Growth of *C. beijerinckii* 8052 was estimated using a DU800 spectrophotometer (Beckman Coulter Inc., Brea, CA) to measure the OD_600_. Concentrations of fermentation products–acetate, butyrate, acetone, butanol, and ethanol were measured using a 7890A Agilent Technologies gas chromatograph (Agilent Technologies Inc., Wilmington, DE) equipped with a flame ionization detector (FID) and 30 m (length) × 320 m (internal diameter) × 0.50 m (HP-Innowax film) J × W 19091N-213 capillary column as described previously [50]. Initial and residual furfural concentrations in the growth medium were determined as described previously [7].

### Total RNA purification

The total cellular RNA was purified from 2 mL *C. beijerinckii* 8052 culture. Briefly, *C. beijerinckii* 8052 cell pellets from 2 mL culture aliquots were thawed on ice and re-suspended in the addition of 750 μL RLT buffer™ (Qiagen Inc., Valencia, CA) supplemented with 1% (v/v) β-mercaptoethanol (β- ME). For complete lysis of *C. beijerinckii* 8052, a 2 mL microcentrifuge tube was half filled with 0.1 mm diameter zirconia/silica beads (Biospec, Bartlesville, OK) followed by 750 μL RLT buffer™ supplemented with 1% (v/v) β-ME and then chilling on ice. *C. beijerinckii* 8052 suspension (750 μL) was added to the pre-chilled tube with beads and agitated in a TissueLyser LT (Qiagen Inc., Valencia, CA) for 8 min at a setting of 50 Hz to break the *C. beijerinckii* 8052 cells. Total RNA was purified from homogenized cells using an RNeasy mini kit (Qiagen Inc., Valencia, CA) according to the manufacturer's instructions. RNA quality was analyzed using a Nanochip 2100 bioanalyzer (Agilent Technologies Inc., Wilmington, DE), and RNA concentration was measured by NanoDrop 3300 (Thermo scientific, Wilmington, DE) according to the manufacturer’s instructions.

### Comparative microarray hybridization

The microarray was constructed by MYcroarray Inc. (Ann Arbor, MI) [52,53]. A total of 5,003 *C. beijerinckii* NCIMB 8052 genes capturing about 99.7% of the genome were examined. To minimize error, five identical replicates of each *C. beijerinckii* 8052 probe sequence (45-47mer) were designed and fabricated onto the microarray chip. To enhance hybridization, 10 μg of total RNA was converted to enriched mRNA using a MICROBExpress™ Bacterial mRNA Enrichment Kit (Life Technologies, Grand Island, NY) and following manufacturer’s protocol. About 200 ng of enriched mRNA was converted to complementary RNA (cRNA) using a MessageAmp™ II-Bacteria RNA Amplification Kit (Life Technologies, Grand Island, NY) and following manufacturer’s protocol. Alexa Fluor 555 (Life Technologies, Grand Island, NY) was coupled to cRNA following the manufacturer’s instructions. Removal of unincorporated dye was conducted using RNeasy Mini Columns (Qiagen Inc., Valencia, CA) and following manufacturer’s protocol. About 30 – 50 μL eluate containing ~40 μg labeled cRNA was generated. The resulting dye-coupled cRNA was made up to 60 μL with 2 μL 150 mM ZnSO_4_ and elution buffer to bring the final concentration of ZnSO_4_ to 5 mM; this dilution was followed by incubation (fragmentation) at 75°C for 10 min. Ten micrograms of each fluor-labeled cRNA was hybridized separately to one array, and hybridization was performed for 20 h at 45°C in a hybridization buffer containing 6x SSPE (20X SSPE stock: 3 M NaCl, 20 mM EDTA, 118.2 mM NaH_2_PO_4_ and 81.8 mM Na_2_HPO_4_), 10% de-ionized formamide, 0.01 mg/mL acetylated BSA, 0.01% Tween-20, and 1% control oligos (provided by Mycroarray Inc). After hybridization, the slide was washed with fresh 6x SSPE buffer (24°C) and transferred to fresh 0.5× SSPE buffer prior to drying. The slide was dried immediately by centrifugation at 2000 g for 3 min prior to scanning by an Axon 4000B Scanner (Molecular Devices, Sunnyvale, CA).

### Microarray data analysis

The slide was scanned using an Axon 4000B Scanner set at 5 μm per pixel resolution and 100% laser power. The scanned images were extracted and analyzed using version 6.1.0.4 GenePix Pro Software (Molecular Devices, Sunnyvale, CA). For signal extraction, circular feature indicators (35 μm diameter) were centered over each spot, and the median feature pixel intensity was extracted. Data images were extracted from the center of the spot area (35 μm in diameter), where the sequence fidelity is exceptional, instead of from the larger indicator feature spot diameter area (~60μm). To minimize error due to differences in sample behavior from array to array, a scale factor was created to normalize the signal across all arrays. A scale factor (SF) for each array was calculated as follows:

$$\begin{array}{ll} \mathrm{SF}= & \mu {\left(\mathrm{median}\;\mathrm{pixel}\;\mathrm{signal}\right)}_{\mathrm{control}}/\\ & \mu {\left(\mathrm{median}\;\mathrm{pixel}\;\mathrm{signal}\right)}_{\mathrm{treatment}} \end{array}$$

Trimmed mean was used to generate the final signal value for the five identical probe replicates, which identified the differential expression pattern of each gene on the array. The trimmed mean was calculated by discarding the maximum and minimum adjusted signal within each set of five probe replicates, followed by averaging the adjusted signal values of the remaining three probes. Relative expression levels (gene expression ratio) for each gene were calculated by dividing the signal intensity of the array from the furfural-challenged *C. beijerinckii* 8052 culture by the intensity of the unchallenged control culture. To facilitate a fair comparison of up- and down-regulated genes, fold change was calculated as follows: for genes with an expression ratio ≥1, the fold change is the same as the expression ratio, whereas folds change of genes whose expression ratio is <1 equals the reciprocal of the expression ratio multiplied by −1 [54]. To make data distribution symmetrical, the gene expression ratio was used to calculate the log2 transformation ratio as described previously [55]. Expression patterns were visualized colorimetrically using TreeView (version 1.60). Enrichment analysis of Gene Ontology terms, including biological process, cellular component and molecular function, and KEGG (Kyoto Encyclopedia of Genes and Genomes) enrichment pathway analysis were performed using a DAVID Functional Annotation Bioinformatics Microarray analysis to identify statistically over-represented biological terms [56].

### Microarray data accession number

All protocols related to this microarray platform, which include information on probe sequences and synthesis, labeling, hybridization and scan protocols, and microarray data have been submitted to NCBI’s Gene Expression Omnibus database at http://www.ncbi.nlm.nih.gov/geo/ with GEO accession number GSE42597.

### Real-time quantitative reverse transcription PCR (Q-RT-PCR)

Following microarray analysis, several genes were differentially induced or repressed in response to furfural stress, and several genes were selected for further analysis using Q-RT-PCR to validate microarray results. Briefly, *C. beijerinckii* 8052 cultures were grown anaerobically and challenged with 2–3 g/L furfural during acidogenic and solventogenic growth phases followed by centrifugation to collect cell pellets as described above. Total RNA was purified from cell pellets of furfural-challenged and unchallenged *C. beijerinckii* 8052 cultures and lysed with TissueLyser LT as described above. Genomic DNA was removed from the total RNA isolate using RNase-free DNase (New England Biolabs Inc, Ipswich, MA). Total RNA (2 μg) was reverse transcribed to form first strand cDNA by random hexamer-primed reverse transcription reactions using SuperScript III reverse transcriptase (Life Technologies, Grand Island, NY) following the manufacturer’s instructions. For quantitative reverse transcription polymerase chain reaction (Q-RT-PCR), cDNA, specific primers and GoTaq® qPCR Master Mix containing Bryt™ Green dye (Promega, Madison, WI) were proportionately mixed following manufacturer’s protocol. The forward and reverse gene-specific primers used for amplification of specific genes were synthesized by Eurofins MWG Operon and are listed in Additional file 4: Table S1. The 16S rRNA of *C. beijerinckii* 8052, which was amplified with gene-specific forward (5′- GAA GAA TAC CAG TGG CGA AGG C-3′) and reverse (5′- ATT CAT CGT TTA CGG CGT GGA C-3′) primers, was used as the internal standard. Prior to selection of 16S rRNA as an internal standard, the expression of 16s rRNA of furfural-challenged and unchallenged *C. beijerinckii* 8052 cultures was analyzed and confirmed for constant expression under the reaction condition of the study. The mRNA levels of genes of interest (Additional file 4: Table S1) were quantified by subjecting cDNA to Q-RT-PCR analysis in triplicate samples using a Bio-Rad iCycler continuous fluorescence detection system (Bio-Rad, Hercules, CA). The Q-RT-PCR reaction conditions were as follows: step 1, 95°C for 2 min (hot-start activation), step 2, 95°C for 15 sec (denaturation), step 3, 55°C for 30 sec (annealing and extension), 40 cycles of step 2 and 3, step 4, 95°C for 1 min (denature of PCR product), step 5, 55°C for 1 min (annealing of PCR product), and step 6, heat from 65°C to 95°C with a ramp speed of 1°C per 10 sec, resulting in melting curves. Expression levels of *C. beijerinckii* 8052 genes were quantified by the comparative C_T_ method as previously described [57].

## Abbreviations

ATCC: American Type Culture Collection; DNA: Deoxyribonucleic acid; GC: Gas chromatography; HPLC: High pressure liquid chromatography; NAD+: Nicotinamide adenine dinucleotide; NADH: Nicotinamide adenine dinucleotide, reduced; NADP+: Nicotinamide adenine dinucleotide phosphate; NADPH: Nicotinamide adenine dinucleotide phosphate, reduced; PCR: Polymerase chain reaction; Q-RT-PCR: Quantitative real-time polymerase chain reaction; RNA: Ribonucleic acid.

## Competing interests

The authors declare that they have no competing interests.

## Authors’ contributions

YZ conducted the work presented here, performed data analysis and drafted the manuscript. YZ and TCE contributed to data interpretation, wrote and revised the manuscript. TCE conceived, designed and coordinated the study. All authors read and approved the final manuscript.

## Supplementary Material

Additional file 1: Table S4 Genes up- and down-regulated by more than 3 folds during acidogenic furfural-challenge. Click here for file

Additional file 2: Table S2A Enriched up- and down-regulated Gene Ontology Groups in the experiment of furfural challenge during acidogenesis. Click here for file

Additional file 3: Table S3 Significantly regulated KEGG classifications during furfural challenge experiment. Click here for file

Additional file 4: Table S1 List of 30 genes and sequences of primers used in validation of microarray analysis by Q-RT-PCR. Click here for file

Additional file 5: Table S5 Fold change of solvent production genes according to microarray analysis. Click here for file
