# Supplementary material for: Transcriptional analysis of Clostridium beijerinckii NCIMB 8052 to elucidate role of furfural stress during acetone butanol ethanol fermentation
Source: Biotechnol Biofuels. 2013 May 4;6:66. doi: 10.1186/1754-6834-6-66 (PMC3681630; doi:10.1186/1754-6834-6-66)
Supplement: Additional file 1: Table S4 — Genes up- and down-regulated by more than 3 folds during acidogenic furfural-challenge. [file 1754-6834-6-66-S1.doc]

Table S4A: Genes up-regulated by more than 3 folds during acidogenic furfural-challenge.

| Gene Symbol | Gene Name | Fold Change |
| --- | --- | --- |
| Cbei_0028 | carbamoyl-phosphate synthase, small subunit | 3.36 |
| Cbei_0068 | anaerobic ribonucleoside-triphosphate reductase | 5.47 |
| Cbei_0194 | ribonucleoside-diphosphate reductase, alpha | 16.57 |
| Cbei_0195 | Ribonucleoside-diphosphate reductase | 7.86 |
| Cbei_0310 | electron transfer flavoprotein, alpha/beta-subunit-like protein | 3.82 |
| Cbei_0311 | electron transfer flavoprotein subunit alpha-like protein | 5.18 |
| Cbei_0312 | FAD linked oxidase domain protein | 3.95 |
| Cbei_0328 | co-chaperonin GroES* | 1.68 |
| Cbei_0329 | chaperonin GroEL* | 1.85 |
| Cbei_0349 | nitrogen-fixing NifU domain protein | 6.66 |
| Cbei_0350 | methyl-accepting chemotaxis sensory transducer | 3.17 |
| Cbei_0389 | Glutathione peroxidase | 3.52 |
| Cbei_0464 | conserved membrane protein | 8.99 |
| Cbei_0538 | hypothetical protein | 4.59 |
| Cbei_0795 | 4Fe-4S ferredoxin, iron-sulfur binding domain | 3.36 |
| Cbei_0829 | heat shock protein GrpE* | 2.75 |
| Cbei_0830 | molecular chaperone DnaK* | 1.38 |
| Cbei_0831 | chaperone protein DnaJ* | 1.56 |
| Cbei_0959 | sugar isomerase (SIS) | 3.05 |
| Cbei_0960 | PfkB domain protein | 3.25 |
| Cbei_1000 | aspartate carbamoyltransferase | 3.25 |
| Cbei_1006 | orotate phosphoribosyltransferase | 3.34 |
| Cbei_1061 | hypothetical protein | 5.34 |
| Cbei_1138 | hypothetical protein | 4.48 |
| Cbei_1224 | riboflavin biosynthesis protein RibD | 9.19 |
| Cbei_1225 | riboflavin synthase, alpha subunit | 9.11 |
| Cbei_1226 | GTP cyclohydrolase II | 15.44 |
| Cbei_1227 | Riboflavin synthase | 6.83 |
| Cbei_1254 | ATP-dependent protease La | 3.26 |
| Cbei_1256 | helix-turn-helix domain protein | 3.43 |
| Cbei_1374 | hypothetical protein | 4.14 |
| Cbei_1464 | Alcohol dehydrogenase GroES domain protein | 3.79 |
| Cbei_1724 | glycosyl transferase, family 2 | 5.12 |
| Cbei_1845 | conserved hypothetical protein | 3.17 |
| Cbei_1849 | FeS assembly protein SufB | 4.06 |
| Cbei_1850 | FeS assembly protein SufD | 4.23 |
| Cbei_1852 | SUF system FeS assembly protein, NifU family | 4.93 |
| Cbei_1885 | regulatory protein GntR, HTH | 6.54 |
| Cbei_1887 | hypothetical protein | 6.01 |
| Cbei_1911 | hypothetical protein | 4.35 |
| Cbei_2057 | protein of unknown function DUF1304 | 5.12 |
| Cbei_2058 | DSBA oxidoreductase | 3.09 |
| Cbei_2121 | alpha/beta hydrolase fold | 3.30 |
| Cbei_2222 | MATE efflux family protein | 3.60 |
| Cbei_2422 | peptidase U61, LD-carboxypeptidase A | 3.18 |
| Cbei_2445 | hypothetical protein | 14.00 |
| Cbei_2522 | ribonucleoside-triphosphate reductase, | 10.13 |
| Cbei_2680 | Redoxin domain protein | 3.39 |
| Cbei_2681 | thioredoxin reductase | 3.18 |
| Cbei_2752 | hypothetical protein | 3.02 |
| Cbei_2792 | MazG nucleotide pyrophosphohydrolase | 3.00 |
| Cbei_2838 | transcriptional regulator, MarR family | 3.59 |
| Cbei_2839 | methyl-accepting chemotaxis sensory transducer | 8.10 |
| Cbei_2842 | conserved hypothetical protein | 3.20 |
| Cbei_2843 | conserved hypothetical protein | 10.55 |
| Cbei_2907 | phosphotransferase system PTS, sorbose-specific | 3.45 |
| Cbei_2923 | secretion protein HlyD family protein | 3.26 |
| Cbei_2951 | two component transcriptional regulator, winged | 3.26 |
| Cbei_2998 | hypothetical protein | 3.20 |
| Cbei_3045 | methyl-accepting chemotaxis sensory transducer | 3.28 |
| Cbei_3298 | putative galactoside ABC transporter | 4.15 |
| Cbei_3300 | ABC transporter related | 3.16 |
| Cbei_3354 | NADPH-dependent FMN reductase | 5.06 |
| Cbei_3613 | conserved hypothetical protein | 3.20 |
| Cbei_3614 | conserved hypothetical protein | 3.55 |
| Cbei_3615 | methionine aminopeptidase, type I | 27.08 |
| Cbei_3616 | transcriptional regulator, XRE family | 20.34 |
| Cbei_3693 | ABC transporter related | 7.25 |
| Cbei_3694 | cobalt transport protein | 6.60 |
| Cbei_3695 | cobalamin (vitamin B12) biosynthesis CbiM | 4.86 |
| Cbei_3904 | short-chain dehydrogenase/reductase SDR | 4.15 |
| Cbei_3948 | nitroreductase | 4.71 |
| Cbei_3973 | putative transcriptional regulator, MerR family | 5.43 |
| Cbei_3974 | aldo/keto reductase | 9.04 |
| Cbei_4216 | putative type IV pilin | 3.35 |
| Cbei_4218 | type II secretion system protein E | 4.39 |
| Cbei_4219 | peptidase A24A domain protein | 3.79 |
| Cbei_4835 | 4Fe-4S ferredoxin, iron-sulfur binding domain | 3.62 |

*Heat shock proteins with less than 3-fold changes

Table S4B: Genes down-regulated by more than 3 folds during acidogenic furfural-challenge

| Gene Symbol | Gene Name | Fold Change |
| --- | --- | --- |
| Cbei_0303 | conserved hypothetical protein | -3.42 |
| Cbei_0304 | small multidrug resistance protein | -4.00 |
| Cbei_2167 | hypothetical protein | -3.30 |
| Cbei_2168 | hypothetical protein | -3.14 |
| Cbei_2169 | hypothetical protein | -4.01 |
| Cbei_2170 | hypothetical protein | -5.62 |
| Cbei_2446 | ornithine carbamoyltransferase | -3.62 |
| Cbei_2725 | response regulator receiver sensor signal | -4.66 |
| Cbei_2727 | putative signal transduction protein | -3.19 |
| Cbei_2830 | glycoside hydrolase | -3.47 |
| Cbei_3006 | hydrogenase expression/formation protein HypE | -3.25 |
| Cbei_3277 | class II aldolase/adducin family protein | -5.25 |
| Cbei_3278 | coenzyme A transferase | -3.40 |
| Cbei_3445 | small multidrug resistance protein | -4.24 |
| Cbei_3446 | Radical SAM domain protein | -6.99 |
| Cbei_3447 | solo B3/4 domain (OB-fold DNA/RNA-binding) of | -5.76 |
| Cbei_3587 | 4Fe-4S ferredoxin, iron-sulfur binding domain | -5.92 |
| Cbei_3600 | 4Fe-4S ferredoxin, iron-sulfur binding domain | -7.85 |
| Cbei_3755 | hypothetical protein | -4.88 |
| Cbei_3825 | ABC transporter permease protein | -3.12 |
| Cbei_3871 | PTS system, mannose/fructose/sorbose family, IID | -3.97 |
| Cbei_3872 | PTS system, mannose/fructose/sorbose family, IIC | -3.48 |
| Cbei_4019 | CheA signal transduction histidine kinase | -3.70 |
| Cbei_4090 | GCN5-related N-acetyltransferase | -3.87 |
| Cbei_4273 | MotA/TolQ/ExbB proton channel | -3.80 |
| Cbei_4515 | Argininosuccinate synthase | -5.80 |
| Cbei_4516 | argininosuccinate lyase | -4.48 |
| Cbei_4517 | N-acetyl-gamma-glutamyl-phosphate reductase | -6.81 |
| Cbei_4518 | arginine biosynthesis bifunctional protein ArgJ | -4.64 |
| Cbei_4519 | acetylglutamate kinase | -5.17 |
| Cbei_4520 | acetylornithine and succinylornithine | -7.69 |
| Cbei_4521 | ornithine carbamoyltransferase | -5.40 |
| Cbei_4911 | PTS system, mannose/fructose/sorbose family, IID | -3.70 |
| Cbei_4912 | PTS system, mannose/fructose/sorbose family, IIC | -3.05 |
| Cbei_4913 | PTS system, mannose/fructose/sorbose family, IIB | -3.49 |
| Cbei_4914 | PTS system, mannose/fructose/sorbose family, IIA | -3.52 |

Table S4C: Genes up-regulated by more than 3 folds during solventogenic furfural-challenge

| Gene Symbol | Gene Name | Fold Change |
| --- | --- | --- |
| Cbei_0012 | GCN5-related N-acetyltransferase | 3.19 |
| Cbei_0062 | prolipoprotein diacylglyceryl transferase | 3.47 |
| Cbei_0068 | anaerobic ribonucleoside-triphosphate reductase | 6.14 |
| Cbei_0143 | ribosomal protein L7/L12 | 3.10 |
| Cbei_0194 | ribonucleoside-diphosphate reductase, alpha | 18.08 |
| Cbei_0195 | Ribonucleoside-diphosphate reductase | 9.09 |
| Cbei_0209 | TrpR like protein, YerC/YecD | 3.21 |
| Cbei_0277 | cyclase family protein | 4.26 |
| Cbei_0278 | transcriptional regulator, MarR family | 10.43 |
| Cbei_0279 | methyl-accepting chemotaxis sensory transducer | 12.13 |
| Cbei_0306 | ATPase, P-type (transporting), HAD superfamily, | 4.83 |
| Cbei_0315 | pyruvate formate-lyase | 6.11 |
| Cbei_0316 | glycyl-radical enzyme activating protein family | 6.35 |
| Cbei_0317 | Transaldolase | 3.12 |
| Cbei_0328 | co-chaperonin GroES | 3.34 |
| Cbei_0329 | chaperonin GroEL | 3.84 |
| Cbei_0337 | PTS system, glucitol/sorbitol-specific, IIBC | 9.89 |
| Cbei_0347 | transcriptional regulator, HxlR family | 3.81 |
| Cbei_0348 | transcriptional regulator, MarR family | 6.26 |
| Cbei_0349 | nitrogen-fixing NifU domain protein | 9.72 |
| Cbei_0350 | methyl-accepting chemotaxis sensory transducer | 23.75 |
| Cbei_0368 | polysaccharide deacetylase | 3.15 |
| Cbei_0379 | 2-alkenal reductase | 3.19 |
| Cbei_0460 | 2-deoxy-D-gluconate 3-dehydrogenase | 3.28 |
| Cbei_0461 | PfkB domain protein | 3.39 |
| Cbei_0538 | hypothetical protein | 10.47 |
| Cbei_0645 | ATPase AAA-2 domain protein | 9.66 |
| Cbei_0702 | transcriptional regulator, DeoR family | 3.07 |
| Cbei_0722 | transcriptional regulator, LysR family | 12.15 |
| Cbei_0748 | deoxyribose-phosphate aldolase | 4.17 |
| Cbei_0829 | heat shock protein GrpE | 5.10 |
| Cbei_0830 | molecular chaperone DnaK* | 2.75 |
| Cbei_0831 | chaperone protein DnaJ* | 2.71 |
| Cbei_0868 | DNA topoisomerase | 3.73 |
| Cbei_1045 | protein of unknown function DUF107 | 4.02 |
| Cbei_1046 | band 7 protein | 5.45 |
| Cbei_1047 | hypothetical protein | 3.07 |
| Cbei_1048 | hypothetical protein | 5.21 |
| Cbei_1055 | phosphoribosylaminoimidazole-succinocarboxamide | 3.89 |
| Cbei_1127 | phosphate binding protein | 9.61 |
| Cbei_1128 | phosphate ABC transporter, inner membrane | 8.76 |
| Cbei_1129 | phosphate ABC transporter, inner membrane | 12.27 |
| Cbei_1130 | phosphate ABC transporter, ATPase subunit | 17.68 |
| Cbei_1131 | phosphate uptake regulator, PhoU | 21.44 |
| Cbei_1132 | phosphate uptake regulator, PhoU | 24.09 |
| Cbei_1138 | hypothetical protein | 4.21 |
| Cbei_1153 | Ribulose-phosphate 3-epimerase | 3.96 |
| Cbei_1166 | protein of unknown function DUF177 | 3.19 |
| Cbei_1189 | translation elongation factor Ts | 3.52 |
| Cbei_1207 | ribosomal protein S15 | 3.58 |
| Cbei_1221 | adenylosuccinate lyase | 3.81 |
| Cbei_1241 | conserved hypothetical protein | 13.42 |
| Cbei_1253 | transcriptional regulator, TetR family | 3.69 |
| Cbei_1254 | ATP-dependent protease La | 12.38 |
| Cbei_1256 | helix-turn-helix domain protein | 7.53 |
| Cbei_1257 | precorrin-3B C17-methyltransferase | 3.35 |
| Cbei_1258 | precorrin-6x reductase | 3.55 |
| Cbei_1317 | Histidinol dehydrogenase | 4.82 |
| Cbei_1319 | Imidazoleglycerol-phosphate dehydratase | 4.54 |
| Cbei_1382 | histidine kinase internal region | 4.07 |
| Cbei_1423 | prolipoprotein diacylglyceryl transferase | 4.04 |
| Cbei_1435 | Heavy metal transport/detoxification protein | 4.26 |
| Cbei_1441 | binding-protein-dependent transport systems | 3.03 |
| Cbei_1460 | uncharacterized protein | 8.34 |
| Cbei_1461 | hypothetical protein | 6.20 |
| Cbei_1462 | hypothetical protein | 7.28 |
| Cbei_1572 | tRNA/rRNA methyltransferase (SpoU) | 3.59 |
| Cbei_1581 | UDP-N-acetylmuramoylalanyl-D-glutamyl-2,6-diaminopimelate--D-alanyl-D-alanyl ligase | 3.55 |
| Cbei_1632 | DNA methylase N-4/N-6 domain protein | 3.42 |
| Cbei_1724 | glycosyl transferase family protein | 9.45 |
| Cbei_1725 | glycosyl transferase family protein | 6.57 |
| Cbei_1762 | Extracellular ligand-binding receptor | 3.18 |
| Cbei_1764 | inner-membrane translocator | 4.23 |
| Cbei_1765 | ABC transporter related | 3.88 |
| Cbei_1766 | ABC transporter related | 7.86 |
| Cbei_1767 | Extracellular ligand-binding receptor | 9.15 |
| Cbei_1768 | conserved hypothetical protein | 3.34 |
| Cbei_1832 | Glucuronate isomerase | 3.36 |
| Cbei_1833 | Mannitol dehydrogenase, C-terminal domain | 3.95 |
| Cbei_1845 | conserved hypothetical protein | 4.12 |
| Cbei_1846 | Hemerythrin HHE cation binding domain protein | 3.06 |
| Cbei_1847 | transcriptional regulator, BadM/Rrf2 family | 4.19 |
| Cbei_1848 | FeS assembly ATPase SufC | 34.66 |
| Cbei_1849 | FeS assembly protein SufB | 24.45 |
| Cbei_1850 | FeS assembly protein SufD | 16.67 |
| Cbei_1851 | cysteine desulfurase, SufS subfamily | 27.29 |
| Cbei_1852 | SUF system FeS assembly protein, NifU family | 54.10 |
| Cbei_1853 | pyruvate flavodoxin/ferredoxin oxidoreductase | 3.57 |
| Cbei_1912 | hypothetical protein | 6.12 |
| Cbei_2043 | conserved hypothetical protein | 3.56 |
| Cbei_2057 | protein of unknown function DUF1304 | 80.04 |
| Cbei_2058 | DSBA oxidoreductase | 9.51 |
| Cbei_2063 | phosphoenolpyruvate synthase | 3.84 |
| Cbei_2089 | ABC transporter related | 5.26 |
| Cbei_2108 | regulatory protein, ArsR | 3.94 |
| Cbei_2109 | Arsenical resistance operon trans-acting | 3.11 |
| Cbei_2110 | arsenite-activated ATPase ArsA | 5.11 |
| Cbei_2111 | protein tyrosine phosphatase | 4.45 |
| Cbei_2112 | arsenical-resistance protein | 4.39 |
| Cbei_2145 | ABC transporter related | 4.38 |
| Cbei_2205 | putative transcriptional regulator, GntR family | 3.51 |
| Cbei_2252 | two component transcriptional regulator | 3.74 |
| Cbei_2290 | hypothetical protein | 12.57 |
| Cbei_2306 | phage integrase family protein | 3.72 |
| Cbei_2419 | Polyphosphate kinase | 6.97 |
| Cbei_2440 | conserved hypothetical protein | 16.48 |
| Cbei_2445 | hypothetical protein | 18.03 |
| Cbei_2459 | 2-keto-3-deoxygluconate permease | 3.81 |
| Cbei_2463 | cobalamin synthesis protein, P47K | 3.34 |
| Cbei_2464 | Putative GTPase (G3E family)-like protein | 3.01 |
| Cbei_2522 | ribonucleoside-triphosphate reductase, adenosylcobalamin-dependent | 39.42 |
| Cbei_2524 | Cof-like hydrolase | 4.78 |
| Cbei_2535 | ferrous iron transport protein B | 3.89 |
| Cbei_2538 | 6-pyruvoyl tetrahydrobiopterin synthase, | 3.43 |
| Cbei_2541 | exsB protein | 3.47 |
| Cbei_2542 | GTP cyclohydrolase I | 3.11 |
| Cbei_2570 | hypothetical protein | 5.48 |
| Cbei_2571 | hypothetical protein | 8.80 |
| Cbei_2659 | pseudouridine synthase | 3.22 |
| Cbei_2680 | Redoxin domain protein | 11.04 |
| Cbei_2681 | thioredoxin reductase | 9.82 |
| Cbei_2721 | putative transcriptional regulator, MerR family | 3.19 |
| Cbei_2792 | MazG nucleotide pyrophosphohydrolase | 3.62 |
| Cbei_2839 | methyl-accepting chemotaxis sensory transducer | 3.19 |
| Cbei_2852 | isochorismatase hydrolase | 3.34 |
| Cbei_2853 | major facilitator superfamily MFS_1 | 3.74 |
| Cbei_2865 | hypothetical protein | 4.03 |
| Cbei_2866 | transcriptional regulator, TetR family | 3.88 |
| Cbei_2877 | transcriptional regulator, TetR family | 4.29 |
| Cbei_2922 | secretion protein HlyD | 3.83 |
| Cbei_2933 | diguanylate cyclase | 4.42 |
| Cbei_2946 | conserved hypothetical protein | 5.44 |
| Cbei_2949 | uncharacterized conserved protein, CotF | 3.19 |
| Cbei_2950 | integral membrane sensor signal transduction | 7.80 |
| Cbei_2951 | two component transcriptional regulator, winged | 13.80 |
| Cbei_2952 | hypothetical protein | 12.78 |
| Cbei_2953 | methyl-accepting chemotaxis sensory transducer | 3.13 |
| Cbei_2982 | hypothetical protein | 3.16 |
| Cbei_2997 | hypothetical protein | 23.24 |
| Cbei_2998 | hypothetical protein | 14.38 |
| Cbei_2999 | UbiA prenyltransferase | 3.44 |
| Cbei_3022 | methyl-accepting chemotaxis sensory transducer | 3.93 |
| Cbei_3043 | hypothetical protein | 4.90 |
| Cbei_3053 | two component transcriptional regulator | 4.61 |
| Cbei_3069 | Rubrerythrin | 5.92 |
| Cbei_3095 | helix-turn-helix- domain containing protein, | 3.67 |
| Cbei_3107 | transcriptional regulator, TetR family | 3.17 |
| Cbei_3127 | phosphopentomutase | 3.80 |
| Cbei_3129 | uridine phosphorylase | 3.61 |
| Cbei_3208 | conserved hypothetical protein | 3.07 |
| Cbei_3241 | hypothetical protein | 3.11 |
| Cbei_3276 | amino acid permease-associated region | 3.49 |
| Cbei_3289 | hypothetical protein | 3.92 |
| Cbei_3298 | putative galactoside ABC transporter | 6.21 |
| Cbei_3299 | ABC transporter related | 4.23 |
| Cbei_3300 | ABC transporter related | 3.73 |
| Cbei_3301 | transcriptional regulator, MarR family | 5.42 |
| Cbei_3303 | diguanylate cyclase/phosphodiesterase | 12.87 |
| Cbei_3307 | beta-lactamase domain protein | 3.70 |
| Cbei_3330 | type I phosphodiesterase/nucleotide | 3.04 |
| Cbei_3331 | ABC transporter related | 3.73 |
| Cbei_3354 | NADPH-dependent FMN reductase | 6.17 |
| Cbei_3369 | L-rhamnose 1-epimerase | 3.89 |
| Cbei_3370 | Ribose/galactose isomerase | 3.61 |
| Cbei_3371 | short-chain dehydrogenase/reductase SDR | 4.22 |
| Cbei_3481 | Methyltransferase type 11 | 3.38 |
| Cbei_3533 | transcriptional regulator, HxlR family | 8.29 |
| Cbei_3535 | protein of unknown function DUF1648 | 3.73 |
| Cbei_3536 | regulatory protein, ArsR | 3.53 |
| Cbei_3549 | transcriptional regulator, XRE family | 5.59 |
| Cbei_3550 | conserved hypothetical protein | 6.48 |
| Cbei_3572 | ApbE family lipoprotein | 7.00 |
| Cbei_3613 | conserved hypothetical protein | 3.05 |
| Cbei_3614 | conserved hypothetical protein | 3.79 |
| Cbei_3615 | methionine aminopeptidase, type I | 17.82 |
| Cbei_3616 | transcriptional regulator, XRE family | 6.69 |
| Cbei_3626 | conserved hypothetical protein | 5.22 |
| Cbei_3627 | conserved hypothetical protein | 3.94 |
| Cbei_3628 | DNA binding domain, excisionase family | 3.50 |
| Cbei_3629 | uncharacterized Fe-S protein | 9.98 |
| Cbei_3630 | acetyl-CoA acetyltransferase | 3.04 |
| Cbei_3631 | transcriptional regulator, LysR family | 6.93 |
| Cbei_3643 | nitroreductase | 6.11 |
| Cbei_3644 | transcriptional regulator, TetR family | 4.45 |
| Cbei_3645 | 4Fe-4S ferredoxin, iron-sulfur binding domain | 3.83 |
| Cbei_3664 | peptidase M15B and M15C, D,D-carboxypeptidase | 4.27 |
| Cbei_3739 | metallophosphoesterase | 4.67 |
| Cbei_3757 | Cupin 2, conserved barrel domain protein | 4.80 |
| Cbei_3895 | NADPH-dependent FMN reductase | 4.00 |
| Cbei_3896 | transcriptional regulator, PadR-like family | 6.72 |
| Cbei_3974 | aldo/keto reductase | 3.87 |
| Cbei_4084 | mannonate dehydratase | 3.74 |
| Cbei_4085 | transcriptional regulator, GntR family | 3.04 |
| Cbei_4086 | methyl-accepting chemotaxis sensory transducer | 3.07 |
| Cbei_4122 | conserved hypothetical protein | 3.83 |
| Cbei_4127 | Dihydroxy-acid dehydratase | 3.10 |
| Cbei_4129 | Transcriptional regulator IclR-like protein | 3.68 |
| Cbei_4186 | sulfate adenylyltransferase, large subunit | 4.91 |
| Cbei_4187 | sulfate adenylyltransferase, small subunit | 3.00 |
| Cbei_4190 | sulfate ABC transporter, ATPase subunit | 7.35 |
| Cbei_4191 | sulfate ABC transporter, inner membrane subunit | 4.72 |
| Cbei_4193 | sulfate ABC transporter, periplasmic | 3.12 |
| Cbei_4199 | isocitrate dehydrogenase, NADP-dependent | 3.36 |
| Cbei_4235 | cellulose synthase subunit domain | 3.17 |
| Cbei_4287 | Integrase, catalytic region | 3.30 |
| Cbei_4336 | aminotransferase, class V | 6.03 |
| Cbei_4337 | thiamine pyrophosphate protein domain protein | 7.14 |
| Cbei_4338 | cytidyltransferase-related domain | 5.07 |
| Cbei_4339 | Nucleotidyl transferase | 7.02 |
| Cbei_4362 | hypothetical protein | 3.47 |
| Cbei_4408 | carboxynorspermidine decarboxylase | 3.38 |
| Cbei_4439 | biotin and thiamin synthesis associated | 4.91 |
| Cbei_4440 | uncharacterized protein | 9.46 |
| Cbei_4444 | binding-protein-dependent transport systems | 3.21 |
| Cbei_4593 | hypothetical protein | 4.40 |
| Cbei_4669 | glycoside hydrolase, family 1 | 3.48 |
| Cbei_4695 | Choline/ethanolamine kinase | 4.94 |
| Cbei_4734 | galactoside O-acetyltransferase | 3.46 |
| Cbei_4883 | DEAD_2 domain protein | 3.02 |
| Cbei_4992 | hypothetical protein | 3.06 |
| Cbei_5023 | hypothetical protein | 3.81 |
| Cbei_5026 | two component transcriptional regulator, winged | 3.78 |
| Cbei_5031 | sigma-54 factor, interaction domain-containing | 5.91 |
| Cbei_5043 | inner-membrane translocator | 8.00 |
| Cbei_5044 | inner-membrane translocator | 7.90 |
| Cbei_5045 | ABC transporter related | 9.36 |
| Cbei_5046 | ABC transporter related | 10.20 |
| Cbei_5074 | Adenylosuccinate synthase | 3.29 |

*Heat shock proteins with less than 3-fold changes.

Table S4D: Genes down-regulated by more than 3 folds during solventogenic furfural-challenge

| Gene Symbol | Gene Name | Fold Change |
| --- | --- | --- |
| Cbei_0021 | ABC transporter related | -5.77 |
| Cbei_0028 | carbamoyl-phosphate synthase, small subunit | -3.83 |
| Cbei_0029 | carbamoyl-phosphate synthase, large subunit | -3.09 |
| Cbei_0030 | hypothetical protein | -5.08 |
| Cbei_0031 | metal dependent phosphohydrolase | -4.04 |
| Cbei_0036 | Peptidoglycan-binding domain 1 protein | -3.09 |
| Cbei_0057 | 3D domain protein | -4.04 |
| Cbei_0073 | glycosyl transferase, family 2 | -3.84 |
| Cbei_0074 | metal dependent phosphohydrolase | -7.25 |
| Cbei_0075 | phosphoserine aminotransferase | -3.21 |
| Cbei_0106 | glycyl-tRNA synthetase | -6.94 |
| Cbei_0130 | prolyl-tRNA synthetase | -3.26 |
| Cbei_0236 | efflux transporter, RND family, MFP subunit | -7.12 |
| Cbei_0237 | ABC transporter related | -5.31 |
| Cbei_0238 | protein of unknown function DUF214 | -7.42 |
| Cbei_0247 | serine O-acetyltransferase | -5.93 |
| Cbei_0269 | Accessory gene regulator B | -7.37 |
| Cbei_0281 | hypothetical protein | -13.38 |
| Cbei_0282 | hypothetical protein | -15.78 |
| Cbei_0283 | ABC transporter related | -16.53 |
| Cbei_0284 | hypothetical protein | -12.13 |
| Cbei_0287 | methyl-accepting chemotaxis sensory transducer | -3.09 |
| Cbei_0310 | Electron transfer flavoprotein, | -3.07 |
| Cbei_0336 | PTS system, glucitol/sorbitol-specific, IIC | -3.30 |
| Cbei_0338 | Transaldolase | -3.73 |
| Cbei_0342 | protein of unknown function DUF188 | -3.37 |
| Cbei_0382 | glycosyl transferase, group 1 | -3.01 |
| Cbei_0420 | hypothetical protein | -3.87 |
| Cbei_0437 | peptidase M50 | -4.57 |
| Cbei_0444 | glutamine synthetase, catalytic region | -5.96 |
| Cbei_0463 | hemerythrin-like metal-binding protein | -4.58 |
| Cbei_0487 | NLP/P60 protein | -7.81 |
| Cbei_0503 | S-layer domain protein domain | -4.78 |
| Cbei_0519 | Serine-type D-Ala-D-Ala carboxypeptidase | -3.39 |
| Cbei_0530 | sugar isomerase (SIS) | -4.20 |
| Cbei_0554 | carbon starvation protein CstA | -12.64 |
| Cbei_0572 | CDP-diacylglycerol--serine | -3.10 |
| Cbei_0583 | membrane protein | -3.00 |
| Cbei_0591 | glycoside hydrolase, family 25 | -3.27 |
| Cbei_0593 | tRNA/rRNA methyltransferase (SpoU) | -3.83 |
| Cbei_0594 | degV family protein | -4.49 |
| Cbei_0595 | RNA polymerase, sigma 54 subunit, RpoN | -3.29 |
| Cbei_0608 | heavy metal transport/detoxification protein | -3.04 |
| Cbei_0642 | putative transcriptional regulator, XRE family | -4.31 |
| Cbei_0643 | TPR repeat-containing protein | -4.20 |
| Cbei_0652 | putative tryptophan transport protein | -8.07 |
| Cbei_0656 | deoxyuridine 5'-triphosphate nucleotidohydrolase | -3.81 |
| Cbei_0659 | signal transduction histidine kinase regulating | -3.83 |
| Cbei_0665 | methyl-accepting chemotaxis sensory transducer | -4.18 |
| Cbei_0674 | NAD-dependent aldehyde dehydrogenase-like | -10.50 |
| Cbei_0675 | Coenzyme F390 synthetase-like protein | -11.86 |
| Cbei_0677 | drug resistance transporter, EmrB/QacA | -4.29 |
| Cbei_0681 | Thioesterase | -9.18 |
| Cbei_0682 | hypothetical protein | -8.80 |
| Cbei_0683 | Radical SAM domain protein | -8.57 |
| Cbei_0684 | AMP-dependent synthetase and ligase | -5.12 |
| Cbei_0685 | Alcohol dehydrogenase GroES domain protein | -5.74 |
| Cbei_0686 | AMP-dependent synthetase and ligase | -14.79 |
| Cbei_0687 | 4'-phosphopantetheinyl transferase | -5.48 |
| Cbei_0688 | beta-lactamase domain protein | -8.65 |
| Cbei_0689 | hypothetical protein | -5.28 |
| Cbei_0690 | Radical SAM domain protein | -6.12 |
| Cbei_0691 | acyl-ACP thioesterase | -4.32 |
| Cbei_0692 | phospho-2-dehydro-3-deoxyheptonate aldolase | -5.68 |
| Cbei_0700 | glycoside hydrolase, family 1 | -3.19 |
| Cbei_0719 | Integrase, catalytic region | -3.56 |
| Cbei_0727 | aldehyde dehydrogenase | -3.32 |
| Cbei_0761 | ribose 5-phosphate isomerase | -3.16 |
| Cbei_0796 | UspA domain protein | -3.60 |
| Cbei_0804 | methyl-accepting chemotaxis sensory transducer | -17.58 |
| Cbei_0807 | integral membrane sensor signal transduction | -4.11 |
| Cbei_0809 | TspO and MBR like protein | -3.40 |
| Cbei_0851 | putative deoxyguanosinetriphosphate | -5.04 |
| Cbei_0852 | DNA primase | -5.64 |
| Cbei_0854 | protein of unknown function DUF633 | -6.36 |
| Cbei_0859 | peptidase C26 | -4.20 |
| Cbei_0863 | Alanine racemase | -7.63 |
| Cbei_0864 | 4-alpha-glucanotransferase | -8.50 |
| Cbei_0865 | glycogen/starch/alpha-glucan phosphorylase | -6.51 |
| Cbei_0869 | short-chain dehydrogenase/reductase SDR | -3.10 |
| Cbei_0871 | ammonium transporter | -9.50 |
| Cbei_0874 | transferase hexapeptide repeat containing | -3.72 |
| Cbei_0875 | methyl-accepting chemotaxis sensory transducer | -8.09 |
| Cbei_0894 | hypothetical protein | -4.18 |
| Cbei_0902 | hypothetical protein | -5.52 |
| Cbei_0904 | hypothetical protein | -4.01 |
| Cbei_0905 | hypothetical protein | -3.57 |
| Cbei_0915 | phage-like element pbsx protein XkdM | -5.52 |
| Cbei_0936 | LrgA family protein | -5.13 |
| Cbei_0937 | LrgB family protein | -4.61 |
| Cbei_0941 | Serine-type D-Ala-D-Ala carboxypeptidase | -3.34 |
| Cbei_0958 | PTS system mannose/fructose/sorbose family IID | -3.63 |
| Cbei_0995 | Tetratricopeptide TPR_2 repeat protein | -7.29 |
| Cbei_0996 | mannose-6-phosphate isomerase, class I | -3.77 |
| Cbei_0999 | beta-lactamase, putative | -3.15 |
| Cbei_1000 | aspartate carbamoyltransferase | -5.12 |
| Cbei_1001 | aspartate transcarbamylase regulatory subunit | -5.85 |
| Cbei_1018 | peptidase M50 | -3.48 |
| Cbei_1019 | arginyl-tRNA synthetase | -3.33 |
| Cbei_1020 | protein of unknown function UPF0118 | -3.31 |
| Cbei_1030 | conserved hypothetical protein | -3.76 |
| Cbei_1034 | asparagine synthase (glutamine-hydrolyzing) | -4.21 |
| Cbei_1037 | protein of unknown function DUF327 | -3.73 |
| Cbei_1042 | hypothetical protein | -3.82 |
| Cbei_1068 | 3-oxoacyl-(acyl-carrier-protein) synthase III | -3.21 |
| Cbei_1086 | hypothetical protein | -4.29 |
| Cbei_1160 | pantetheine-phosphate adenylyltransferase | -4.81 |
| Cbei_1184 | Mg chelatase, subunit ChlI | -3.26 |
| Cbei_1218 | Stage V sporulation protein S | -3.79 |
| Cbei_1234 | histidine kinase internal region | -4.45 |
| Cbei_1277 | adenine deaminase | -5.21 |
| Cbei_1278 | conserved hypothetical protein | -4.74 |
| Cbei_1279 | Homoserine O-succinyltransferase | -3.41 |
| Cbei_1280 | putative sodium-glucose/galactose cotransporter | -4.65 |
| Cbei_1297 | hypothetical protein | -4.26 |
| Cbei_1302 | RNA polymerase, sigma-24 subunit, ECF subfamily | -3.35 |
| Cbei_1336 | ferric uptake regulator, Fur family | -3.05 |
| Cbei_1338 | putative nicotinate phosphoribosyltransferase | -3.14 |
| Cbei_1344 | hypothetical protein | -5.34 |
| Cbei_1345 | hypothetical protein | -4.09 |
| Cbei_1346 | hypothetical protein | -9.32 |
| Cbei_1383 | SSS sodium solute transporter superfamily | -3.82 |
| Cbei_1411 | hypothetical protein | -3.04 |
| Cbei_1438 | conserved hypothetical protein | -3.16 |
| Cbei_1454 | peptidase S8 and S53, subtilisin, kexin, | -3.35 |
| Cbei_1458 | pyruvate flavodoxin/ferredoxin oxidoreductase | -3.56 |
| Cbei_1470 | Extradiol ring-cleavage dioxygenase, class III | -4.38 |
| Cbei_1486 | protein of unknown function DUF6, transmembrane | -5.63 |
| Cbei_1508 | hypothetical protein | -3.54 |
| Cbei_1516 | ABC transporter related | -3.58 |
| Cbei_1517 | uncharacterized membrane protein, putative | -4.76 |
| Cbei_1567 | conserved hypothetical protein | -5.04 |
| Cbei_1575 | MraZ protein | -4.14 |
| Cbei_1576 | S-adenosyl-methyltransferase MraW | -3.59 |
| Cbei_1577 | conserved hypothetical protein | -3.02 |
| Cbei_1578 | stage V sporulation protein D | -3.09 |
| Cbei_1579 | stage V sporulation protein D | -3.64 |
| Cbei_1590 | protein of unknown function YGGT | -3.07 |
| Cbei_1598 | transcriptional regulator, RpiR family | -6.13 |
| Cbei_1614 | hypothetical protein | -4.08 |
| Cbei_1621 | RecT protein | -4.44 |
| Cbei_1636 | conserved hypothetical protein | -4.12 |
| Cbei_1638 | hypothetical protein | -3.07 |
| Cbei_1643 | hypothetical protein | -3.48 |
| Cbei_1645 | hypothetical protein | -4.98 |
| Cbei_1646 | hypothetical protein | -3.42 |
| Cbei_1647 | hypothetical protein | -5.55 |
| Cbei_1648 | hypothetical protein | -3.25 |
| Cbei_1652 | SH3, type 3 domain protein | -3.07 |
| Cbei_1669 | hypothetical protein | -3.28 |
| Cbei_1675 | putative cell wall binding repeat-containing | -6.63 |
| Cbei_1680 | hypothetical protein | -3.52 |
| Cbei_1684 | hypothetical protein | -3.83 |
| Cbei_1713 | hemerythrin-like metal-binding protein | -5.66 |
| Cbei_1714 | hypothetical protein | -4.61 |
| Cbei_1715 | uracil-xanthine permease | -7.95 |
| Cbei_1723 | methyl-accepting chemotaxis sensory transducer | -4.49 |
| Cbei_1735 | hypothetical protein | -3.04 |
| Cbei_1770 | major facilitator superfamily MFS_1 | -6.87 |
| Cbei_1771 | nitrite and sulphite reductase 4Fe-4S region | -6.29 |
| Cbei_1774 | protein of unknown function DUF47 | -4.33 |
| Cbei_1775 | phosphate transporter | -7.66 |
| Cbei_1785 | HIRAN | -3.91 |
| Cbei_1810 | conserved hypothetical protein | -3.53 |
| Cbei_1863 | hypothetical protein | -4.06 |
| Cbei_1882 | hypothetical protein | -3.11 |
| Cbei_1895 | protein of unknown function DUF477 | -3.99 |
| Cbei_1902 | hypothetical protein | -3.19 |
| Cbei_1916 | protein of unknown function DUF541 | -4.48 |
| Cbei_1942 | dihydropyrimidinase | -3.28 |
| Cbei_1965 | ABC transporter related | -5.53 |
| Cbei_1966 | molybdate ABC transporter, inner membrane | -5.68 |
| Cbei_1970 | dihydropyrimidinase | -3.19 |
| Cbei_1981 | uracil-xanthine permease | -3.13 |
| Cbei_1982 | aldehyde oxidase and xanthine dehydrogenase, | -3.23 |
| Cbei_1992 | molybdenum ABC transporter, periplasmic | -3.94 |
| Cbei_2004 | nitrogenase MoFe cofactor biosynthesis protein | -4.48 |
| Cbei_2008 | Radical SAM domain protein | -6.58 |
| Cbei_2053 | ApbE family lipoprotein | -3.74 |
| Cbei_2054 | transcriptional regulator, LysR family | -6.19 |
| Cbei_2061 | ammonium transporter | -6.39 |
| Cbei_2069 | manganese containing catalase | -3.61 |
| Cbei_2070 | spore coat peptide assembly protein CotJB | -3.27 |
| Cbei_2071 | hypothetical protein | -3.17 |
| Cbei_2114 | IMP dehydrogenase | -7.24 |
| Cbei_2126 | aspartyl-tRNA synthetase | -6.08 |
| Cbei_2127 | glutamyl-tRNA(Gln) amidotransferase, C subunit | -5.80 |
| Cbei_2128 | glutamyl-tRNA(Gln) amidotransferase, A subunit | -4.46 |
| Cbei_2129 | glutamyl-tRNA(Gln) amidotransferase, B subunit | -3.84 |
| Cbei_2143 | transcriptional regulator, LysR family | -4.12 |
| Cbei_2160 | multi-sensor hybrid histidine kinase | -7.57 |
| Cbei_2165 | hemerythrin-like metal-binding protein | -15.15 |
| Cbei_2166 | hypothetical protein | -14.33 |
| Cbei_2167 | hypothetical protein | -7.76 |
| Cbei_2168 | hypothetical protein | -13.71 |
| Cbei_2169 | hypothetical protein | -28.29 |
| Cbei_2170 | hypothetical protein | -20.00 |
| Cbei_2196 | PTS system mannose/fructose/sorbose family IID | -4.27 |
| Cbei_2212 | Integral membrane protein TerC | -7.54 |
| Cbei_2213 | peptidase M48, Ste24p | -5.79 |
| Cbei_2217 | phage replisome organizer, putative | -3.30 |
| Cbei_2226 | protein of unknown function DUF6, transmembrane | -7.02 |
| Cbei_2251 | SNARE associated Golgi protein | -3.64 |
| Cbei_2260 | hypothetical protein | -3.03 |
| Cbei_2261 | Lytic transglycosylase, catalytic | -3.68 |
| Cbei_2321 | alpha,alpha-phosphotrehalase | -3.34 |
| Cbei_2346 | hypothetical protein | -3.37 |
| Cbei_2348 | hypothetical protein | -3.05 |
| Cbei_2359 | Monosaccharide-transporting ATPase | -3.32 |
| Cbei_2379 | response regulator receiver protein | -5.05 |
| Cbei_2424 | diguanylate cyclase/phosphodiesterase | -3.40 |
| Cbei_2426 | uracil-xanthine permease | -6.36 |
| Cbei_2488 | conserved hypothetical protein | -4.21 |
| Cbei_2489 | protein of unknown function DUF523 | -4.47 |
| Cbei_2490 | Rubrerythrin | -3.47 |
| Cbei_2506 | hypothetical protein | -4.66 |
| Cbei_2516 | response regulator receiver protein | -3.88 |
| Cbei_2518 | aldehyde dehydrogenase | -3.07 |
| Cbei_2563 | RNA chaperone Hfq | -3.56 |
| Cbei_2585 | glycosyl transferase, group 1 | -3.52 |
| Cbei_2591 | glycosyl transferase, family 2 | -4.20 |
| Cbei_2598 | amine oxidase | -3.93 |
| Cbei_2605 | hypothetical protein | -3.25 |
| Cbei_2606 | hypothetical protein | -4.02 |
| Cbei_2635 | hypothetical protein | -3.40 |
| Cbei_2637 | hypothetical protein | -4.61 |
| Cbei_2638 | pentapeptide repeat protein | -3.20 |
| Cbei_2639 | hypothetical protein | -3.38 |
| Cbei_2644 | conserved hypothetical protein | -3.26 |
| Cbei_2645 | ABC transporter related | -3.23 |
| Cbei_2656 | MATE efflux family protein | -3.53 |
| Cbei_2663 | PTS system lactose/cellobiose-specific transporter subunit IIA | -3.35 |
| Cbei_2675 | coagulation factor 5/8 type domain protein | -17.42 |
| Cbei_2725 | response regulator receiver sensor signal | -17.21 |
| Cbei_2726 | response regulator receiver protein | -10.32 |
| Cbei_2727 | putative signal transduction protein | -19.91 |
| Cbei_2728 | PAS/PAC sensor signal transduction histidine | -11.54 |
| Cbei_2739 | putative sugar-specific permease, SgaT/UlaA | -13.65 |
| Cbei_2740 | phosphotransferase system, lactose/cellobiose-specific IIB subunit | -7.84 |
| Cbei_2741 | putative PTS IIA-like nitrogen-regulatory | -7.42 |
| Cbei_2745 | zinc finger, SWIM domain protein | -4.60 |
| Cbei_2746 | VWA containing CoxE family protein | -4.90 |
| Cbei_2747 | hypothetical protein | -3.41 |
| Cbei_2748 | ATPase associated with various cellular | -4.57 |
| Cbei_2749 | conserved hypothetical membrane spanning | -3.95 |
| Cbei_2750 | molybdate metabolism regulator | -5.05 |
| Cbei_2751 | hypothetical protein | -4.12 |
| Cbei_2753 | iron-containing alcohol dehydrogenase | -3.49 |
| Cbei_2761 | conserved hypothetical protein | -4.00 |
| Cbei_2787 | methyl-accepting chemotaxis sensory transducer | -9.14 |
| Cbei_2826 | Carbohydrate-binding family V/XII | -3.44 |
| Cbei_2828 | glycoside hydrolase, family 16 | -5.46 |
| Cbei_2830 | glycoside hydrolase, family 18 | -12.47 |
| Cbei_2831 | Carbohydrate-binding family V/XII | -6.97 |
| Cbei_2888 | nitroreductase | -3.63 |
| Cbei_2907 | phosphotransferase system PTS, sorbose-specific | -4.36 |
| Cbei_2916 | NHL repeat containing protein | -3.17 |
| Cbei_2920 | integral membrane sensor signal transduction | -3.16 |
| Cbei_2954 | Cupin 2, conserved barrel domain protein | -3.32 |
| Cbei_2957 | cation diffusion facilitator family transporter | -4.24 |
| Cbei_2980 | multicopper oxidase, type 3 | -3.21 |
| Cbei_3007 | hydrogenase formation HypD protein | -3.00 |
| Cbei_3008 | hydrogenase assembly chaperone hypC/hupF | -3.64 |
| Cbei_3010 | conserved hypothetical protein, CF-29 family | -3.29 |
| Cbei_3014 | hybrid cluster protein | -3.40 |
| Cbei_3018 | beta-lactamase domain protein | -4.93 |
| Cbei_3023 | conserved protein | -3.79 |
| Cbei_3044 | hypothetical protein | -3.60 |
| Cbei_3072 | conserved hypothetical protein | -3.07 |
| Cbei_3076 | diguanylate cyclase | -3.58 |
| Cbei_3088 | iron dependent repressor | -3.30 |
| Cbei_3116 | Glutaredoxin-related protein | -3.34 |
| Cbei_3118 | amino acid permease-associated region | -10.12 |
| Cbei_3119 | methyl-accepting chemotaxis sensory transducer | -3.78 |
| Cbei_3164 | MATE efflux family protein | -3.69 |
| Cbei_3168 | Phosphoglycerate mutase | -4.45 |
| Cbei_3169 | multi-sensor signal transduction histidine | -4.94 |
| Cbei_3170 | conserved hypothetical protein | -4.86 |
| Cbei_3171 | Accessory gene regulator B | -3.67 |
| Cbei_3178 | Lysine exporter protein (LYSE/YGGA) | -4.51 |
| Cbei_3197 | glycosyltransferase, MGT family | -14.45 |
| Cbei_3201 | phage replisome organizer, putative | -3.73 |
| Cbei_3202 | conserved hypothetical protein | -5.87 |
| Cbei_3206 | threonyl-tRNA synthetase | -3.58 |
| Cbei_3256 | hypothetical protein | -3.84 |
| Cbei_3257 | Rubrerythrin | -3.21 |
| Cbei_3268 | Holin, phage phi LC3 | -3.61 |
| Cbei_3269 | conserved hypothetical protein | -4.79 |
| Cbei_3278 | coenzyme A transferase | -4.41 |
| Cbei_3288 | metal dependent phosphohydrolase | -3.43 |
| Cbei_3343 | D-galactose-binding periplasmic protein | -3.26 |
| Cbei_3350 | Citrate transporter | -3.27 |
| Cbei_3352 | sigma54 specific transcriptional regulator, Fis | -3.62 |
| Cbei_3356 | methyl-accepting chemotaxis sensory transducer | -3.58 |
| Cbei_3392 | hypothetical protein | -3.14 |
| Cbei_3393 | phage-like element pbsx protein XkdQ | -3.63 |
| Cbei_3395 | Phage-related protein | -3.05 |
| Cbei_3397 | phage-like element pbsx protein XkdM | -3.13 |
| Cbei_3399 | hypothetical protein | -3.87 |
| Cbei_3407 | putative cell wall binding repeat-containing | -7.28 |
| Cbei_3409 | hypothetical protein | -3.14 |
| Cbei_3425 | hypothetical protein | -6.63 |
| Cbei_3444 | hypothetical protein | -3.48 |
| Cbei_3467 | putative transcriptional regulator, MerR family | -5.24 |
| Cbei_3468 | flavodoxin | -3.58 |
| Cbei_3469 | histidine kinase | -3.34 |
| Cbei_3470 | domain of unknown function DUF1745 | -9.41 |
| Cbei_3471 | putative signal transduction protein | -4.04 |
| Cbei_3472 | oxidoreductase FAD/NAD(P)-binding domain | -3.38 |
| Cbei_3473 | CDP-alcohol phosphatidyltransferase | -4.28 |
| Cbei_3486 | methyl-accepting chemotaxis sensory transducer | -4.82 |
| Cbei_3493 | putative transcriptional regulator, MerR family | -3.19 |
| Cbei_3495 | beta-lactamase domain protein | -3.44 |
| Cbei_3544 | 4Fe-4S ferredoxin, iron-sulfur binding domain | -3.01 |
| Cbei_3584 | alpha/beta hydrolase fold | -3.61 |
| Cbei_3587 | 4Fe-4S ferredoxin, iron-sulfur binding domain | -6.72 |
| Cbei_3600 | 4Fe-4S ferredoxin, iron-sulfur binding domain | -4.98 |
| Cbei_3601 | transcriptional regulator, TetR family | -3.86 |
| Cbei_3638 | Lytic transglycosylase, catalytic | -3.35 |
| Cbei_3671 | methyl-accepting chemotaxis sensory transducer | -13.47 |
| Cbei_3672 | dual specificity protein phosphatase | -4.72 |
| Cbei_3680 | hypothetical protein | -4.32 |
| Cbei_3681 | hypothetical protein | -7.90 |
| Cbei_3682 | hypothetical protein | -3.63 |
| Cbei_3683 | cell wall-associated hydrolase-like protein | -5.24 |
| Cbei_3684 | AAA ATPase | -9.83 |
| Cbei_3685 | hypothetical protein | -5.89 |
| Cbei_3686 | hypothetical protein | -6.69 |
| Cbei_3688 | hypothetical protein | -3.46 |
| Cbei_3713 | protein of unknown function DUF1540 | -3.10 |
| Cbei_3755 | hypothetical protein | -39.58 |
| Cbei_3771 | glycoside hydrolase, family 3 domain protein | -3.84 |
| Cbei_3787 | hypothetical protein | -3.27 |
| Cbei_3788 | RNA polymerase, sigma-24 subunit, ECF subfamily | -3.74 |
| Cbei_3789 | membrane protein | -6.40 |
| Cbei_3798 | formate dehydrogenase family accessory protein | -3.52 |
| Cbei_3836 | peptidase C1A, papain | -3.01 |
| Cbei_3838 | GCN5-related N-acetyltransferase | -3.17 |
| Cbei_3871 | PTS system, mannose/fructose/sorbose family, IID | -3.18 |
| Cbei_3872 | PTS system, mannose/fructose/sorbose family, IIC | -3.52 |
| Cbei_3879 | binding-protein-dependent transport systems | -6.47 |
| Cbei_3890 | Alcohol dehydrogenase GroES domain protein | -6.20 |
| Cbei_3891 | aminotransferase class-III | -5.63 |
| Cbei_3912 | VanZ family protein | -4.33 |
| Cbei_3934 | hypothetical protein | -3.33 |
| Cbei_3939 | D-galactose-binding periplasmic protein | -3.33 |
| Cbei_3940 | GCN5-related N-acetyltransferase | -3.88 |
| Cbei_3942 | GCN5-related N-acetyltransferase | -3.08 |
| Cbei_3943 | GCN5-related N-acetyltransferase | -3.14 |
| Cbei_3958 | hypothetical protein | -3.04 |
| Cbei_3961 | methyl-accepting chemotaxis sensory transducer | -3.80 |
| Cbei_3964 | CHAP domain containing protein | -11.31 |
| Cbei_3968 | TPR repeat-containing protein | -5.42 |
| Cbei_3986 | SCP-like extracellular | -12.82 |
| Cbei_3989 | Beta-ketoacyl synthase-like protein | -3.52 |
| Cbei_3990 | transcription activator, effector binding | -3.38 |
| Cbei_3991 | hypothetical protein | -3.40 |
| Cbei_3994 | conserved hypothetical protein | -3.45 |
| Cbei_4007 | 2-keto-3-deoxygluconate permease | -4.09 |
| Cbei_4015 | response regulator receiver protein | -3.77 |
| Cbei_4016 | histidine kinase | -6.92 |
| Cbei_4019 | CheA signal transduction histidine kinase | -15.87 |
| Cbei_4020 | response regulator receiver protein | -17.15 |
| Cbei_4032 | Beta-ketoacyl-acyl-carrier-protein synthase I | -3.99 |
| Cbei_4035 | putative transcriptional regulator, GntR family | -4.90 |
| Cbei_4041 | pyruvate ferredoxin/flavodoxin oxidoreductase, | -3.69 |
| Cbei_4048 | microcompartments protein | -6.14 |
| Cbei_4049 | conserved hypothetical protein | -3.78 |
| Cbei_4058 | microcompartments protein | -3.52 |
| Cbei_4078 | periplasmic binding protein/LacI transcriptional | -4.62 |
| Cbei_4161 | methyl-accepting chemotaxis sensory transducer | -3.81 |
| Cbei_4168 | integral membrane sensor signal transduction | -3.78 |
| Cbei_4169 | two component transcriptional regulator, winged | -3.02 |
| Cbei_4170 | polar amino acid ABC transporter, inner membrane | -6.02 |
| Cbei_4171 | polar amino acid ABC transporter, inner membrane | -4.66 |
| Cbei_4172 | extracellular solute-binding protein, family 3 | -3.72 |
| Cbei_4173 | ABC transporter related | -7.92 |
| Cbei_4175 | signal transduction histidine kinase regulating | -3.77 |
| Cbei_4183 | CheA signal transduction histidine kinase | -6.94 |
| Cbei_4184 | putative CheW protein | -4.42 |
| Cbei_4224 | putative transcriptional regulator, GntR family | -3.82 |
| Cbei_4228 | hypoxanthine phosphoribosyltransferase | -4.30 |
| Cbei_4229 | Sporulation protein YunB | -3.20 |
| Cbei_4254 | flagellar biosynthetic protein FlhB | -3.35 |
| Cbei_4266 | Flagellar biosynthesis/type III secretory | -3.27 |
| Cbei_4267 | flagellar motor switch protein FliG | -4.33 |
| Cbei_4268 | flagellar M-ring protein FliF | -3.94 |
| Cbei_4270 | flagellar basal-body rod protein FlgC | -3.31 |
| Cbei_4273 | MotA/TolQ/ExbB proton channel | -4.76 |
| Cbei_4274 | flagellin domain protein | -3.37 |
| Cbei_4275 | hypothetical protein | -4.00 |
| Cbei_4284 | protein of unknown function DUF115 | -3.57 |
| Cbei_4289 | flagellin domain protein | -3.90 |
| Cbei_4290 | conserved hypothetical protein | -3.82 |
| Cbei_4291 | flagellar hook-associated 2 domain protein | -3.25 |
| Cbei_4292 | flagellar protein FliS | -4.41 |
| Cbei_4295 | carbon storage regulator, CsrA | -4.96 |
| Cbei_4296 | protein of unknown function DUF180 | -5.16 |
| Cbei_4297 | flagellar hook-associated protein 3 | -4.31 |
| Cbei_4307 | CheA signal transduction histidine kinase | -3.09 |
| Cbei_4309 | response regulator receiver modulated CheB | -3.01 |
| Cbei_4314 | HAD family hydrolase | -3.27 |
| Cbei_4329 | hypothetical protein | -6.13 |
| Cbei_4394 | hypothetical protein | -4.08 |
| Cbei_4396 | hypothetical protein | -3.82 |
| Cbei_4397 | Molecular chaperone-like protein | -3.00 |
| Cbei_4398 | hypothetical protein | -5.05 |
| Cbei_4399 | hypothetical protein | -3.81 |
| Cbei_4400 | Serine/threonine protein phosphatase-like protein | -4.77 |
| Cbei_4401 | protein phosphatase 2C domain protein | -6.68 |
| Cbei_4402 | FHA domain containing protein | -4.90 |
| Cbei_4403 | hypothetical protein | -7.07 |
| Cbei_4404 | heat shock protein DnaJ domain protein | -3.99 |
| Cbei_4407 | hypothetical protein | -3.41 |
| Cbei_4418 | RNA-binding region-containing protein (RNP-1) | -3.37 |
| Cbei_4438 | methyl-accepting chemotaxis sensory transducer | -3.74 |
| Cbei_4446 | transcriptional regulator, AraC family | -3.20 |
| Cbei_4466 | methyl-accepting chemotaxis sensory transducer | -4.63 |
| Cbei_4469 | SCP-like extracellular | -4.96 |
| Cbei_4470 | PHP C-terminal domain protein | -3.61 |
| Cbei_4472 | metal dependent phosphohydrolase | -3.97 |
| Cbei_4509 | glycerol kinase | -3.51 |
| Cbei_4513 | protein of unknown function DUF421 | -3.80 |
| Cbei_4514 | conserved hypothetical protein | -4.73 |
| Cbei_4516 | argininosuccinate lyase | -3.41 |
| Cbei_4517 | N-acetyl-gamma-glutamyl-phosphate reductase | -3.12 |
| Cbei_4527 | amino acid permease-associated region | -3.28 |
| Cbei_4532 | PTS system, N-acetylglucosamine-specific IIBC | -17.89 |
| Cbei_4533 | PTS system, glucose subfamily, IIA subunit | -4.08 |
| Cbei_4550 | PfkB domain protein | -3.25 |
| Cbei_4556 | sugar isomerase (SIS) | -3.32 |
| Cbei_4557 | PTS system mannose/fructose/sorbose family IID | -3.38 |
| Cbei_4558 | phosphotransferase system PTS, sorbose-specific | -6.95 |
| Cbei_4559 | PTS system sorbose subfamily IIB component | -3.63 |
| Cbei_4560 | PTS system fructose subfamily IIA component | -5.49 |
| Cbei_4582 | ABC transporter related | -6.55 |
| Cbei_4583 | hypothetical protein | -5.54 |
| Cbei_4584 | ABC transporter related | -7.22 |
| Cbei_4585 | NADPH-dependent FMN reductase | -5.71 |
| Cbei_4586 | Lantibiotic modifying -like protein | -5.06 |
| Cbei_4587 | hypothetical protein | -5.86 |
| Cbei_4590 | leucyl-tRNA synthetase | -4.68 |
| Cbei_4634 | PTS system, lactose/cellobiose family IIC | -4.10 |
| Cbei_4639 | phosphotransferase system, lactose/cellobiose-specific IIB subunit | -3.39 |
| Cbei_4640 | PTS system lactose/cellobiose-specific transporter subunit IIA | -3.37 |
| Cbei_4659 | mannonate dehydratase | -3.87 |
| Cbei_4661 | helix-turn-helix- domain containing protein, | -4.00 |
| Cbei_4678 | binding-protein-dependent transport systems | -3.74 |
| Cbei_4683 | PTS system, lactose/cellobiose family IIC | -4.62 |
| Cbei_4700 | hypothetical protein | -3.23 |
| Cbei_4714 | hypothetical protein | -4.20 |
| Cbei_4718 | putative cell wall binding repeat-containing | -8.67 |
| Cbei_4769 | hypothetical protein | -3.33 |
| Cbei_4778 | hypothetical protein | -3.09 |
| Cbei_4790 | hypothetical protein | -3.30 |
| Cbei_4805 | 6-phospho-beta-glucosidase | -4.76 |
| Cbei_4807 | transcriptional regulator, RpiR family | -3.29 |
| Cbei_4813 | response regulator receiver protein | -4.54 |
| Cbei_4814 | multi-sensor hybrid histidine kinase | -5.72 |
| Cbei_4815 | hypothetical protein | -4.39 |
| Cbei_4816 | hemerythrin-like metal-binding protein | -6.40 |
| Cbei_4817 | hypothetical protein | -5.79 |
| Cbei_4818 | hemerythrin-like metal-binding protein | -4.82 |
| Cbei_4819 | response regulator receiver protein | -3.04 |
| Cbei_4820 | response regulator receiver sensor signal | -5.92 |
| Cbei_4821 | methyl-accepting chemotaxis sensory transducer | -6.06 |
| Cbei_4822 | putative CheW protein | -17.18 |
| Cbei_4823 | methyl-accepting chemotaxis sensory transducer | -7.45 |
| Cbei_4824 | response regulator receiver protein | -18.44 |
| Cbei_4826 | response regulator receiver modulated CheB | -3.38 |
| Cbei_4827 | Protein-glutamate O-methyltransferase | -3.87 |
| Cbei_4828 | methyl-accepting chemotaxis sensory transducer | -4.94 |
| Cbei_4829 | CheA signal transduction histidine kinase | -11.98 |
| Cbei_4832 | methyl-accepting chemotaxis sensory transducer | -6.19 |
| Cbei_4838 | PTS system, glucose subfamily, IIA subunit | -3.43 |
| Cbei_4849 | galactoside ABC transporter periplasmic D-galactose/D-glucose-binding protein | -3.61 |
| Cbei_4877 | conserved hypothetical protein | -3.54 |
| Cbei_4885 | transcriptional regulator, AbrB family | -3.59 |
| Cbei_4908 | glycogen/starch synthase, ADP-glucose type | -3.40 |
| Cbei_4909 | 1,4-alpha-glucan branching enzyme | -3.85 |
| Cbei_4911 | PTS system, mannose/fructose/sorbose family, IID | -3.89 |
| Cbei_4914 | PTS system, mannose/fructose/sorbose family, IIA | -3.36 |
| Cbei_4925 | PAS/PAC sensor signal transduction histidine | -6.60 |
| Cbei_4941 | extracellular solute-binding protein, family 1 | -5.33 |
| Cbei_4943 | response regulator receiver protein | -3.67 |
| Cbei_4960 | pyruvate carboxylase | -3.11 |
| Cbei_4974 | glycoside hydrolase, family 3 domain protein | -3.42 |
| Cbei_4975 | helix-turn-helix- domain containing protein | -5.64 |
| Cbei_4988 | hypothetical protein | -5.84 |
| Cbei_5015 | StbA family protein | -3.11 |
| Cbei_5016 | hypothetical protein | -3.02 |
| Cbei_5032 | metal dependent phosphohydrolase | -3.39 |
| Cbei_5034 | ammonium transporter | -7.29 |
| Cbei_5059 | methyl-accepting chemotaxis sensory transducer | -3.86 |
| Cbei_5065 | protein of unknown function DUF606 | -4.42 |
| Cbei_5066 | beta-lactamase domain protein | -3.47 |
| Cbei_5067 | peptidase S8 and S53, subtilisin, kexin, | -3.01 |
| Cbei_5092 | protein of unknown function DUF1256 | -3.78 |
