# Supplementary material for: Transcriptional analysis of Clostridium beijerinckii NCIMB 8052 to elucidate role of furfural stress during acetone butanol ethanol fermentation
Source: Biotechnol Biofuels. 2013 May 4;6:66. doi: 10.1186/1754-6834-6-66 (PMC3681630; doi:10.1186/1754-6834-6-66)
Supplement: Additional file 2: Table S2A — Enriched up- and down-regulated Gene Ontology Groups in the experiment of furfural challenge during acidogenesis. [file 1754-6834-6-66-S2.doc]

Table S2A: Enriched Up-regulated Gene Ontology Groups in the experiment of furfural challenge during acidogenesis

| GOTERM_Category | GO number | Term | Count | % | P-value |
| --- | --- | --- | --- | --- | --- |
| Biological Process | GO:0016226 | iron-sulfur cluster assembly | 4 | 5.80 | 0.000 |
|  | GO:0031163 | metallo-sulfur cluster assembly | 4 | 5.80 | 0.000 |
|  | GO:0055114 | oxidation reduction | 9 | 13.04 | 0.004 |
|  | GO:0042221 | response to chemical stimulus | 7 | 10.14 | 0.004 |
|  | GO:0051188 | cofactor biosynthetic process | 6 | 8.70 | 0.005 |
|  | GO:0051186 | cofactor metabolic process | 6 | 8.70 | 0.010 |
|  | GO:0006767 | water-soluble vitamin metabolic process | 4 | 5.80 | 0.032 |
|  | GO:0042364 | water-soluble vitamin biosynthetic process | 4 | 5.80 | 0.032 |
|  | GO:0009110 | vitamin biosynthetic process | 4 | 5.80 | 0.035 |
|  | GO:0006766 | vitamin metabolic process | 4 | 5.80 | 0.035 |
|  | GO:0050896 | response to stimulus | 7 | 10.14 | 0.041 |
|  | GO:0006979 | response to oxidative stress | 2 | 2.90 | 0.050 |
|  | GO:0009231 | riboflavin biosynthetic process | 2 | 2.90 | 0.062 |
|  | GO:0042726 | riboflavin and derivative metabolic process | 2 | 2.90 | 0.062 |
|  | GO:0006771 | riboflavin metabolic process | 2 | 2.90 | 0.062 |
|  | GO:0042727 | riboflavin and derivative biosynthetic process | 2 | 2.90 | 0.062 |
| Cellular Component | GO:0005971 | ribonucleoside-diphosphate reductase complex | 2 | 2.90 | 0.022 |
| Molecular Function | GO:0016728 | oxidoreductase activity, acting on CH or CH_2_ groups, disulfide as acceptor | 3 | 4.35 | 0.002 |
|  | GO:0016725 | oxidoreductase activity, acting on CH or CH_2_ groups | 3 | 4.35 | 0.003 |
|  | GO:0016491 | oxidoreductase activity | 13 | 18.84 | 0.005 |
|  | GO:0016209 | antioxidant activity | 3 | 4.35 | 0.018 |
|  | GO:0008998 | ribonucleoside-triphosphate reductase activity | 2 | 2.90 | 0.030 |
|  | GO:0004748 | ribonucleoside-diphosphate reductase activity | 2 | 2.90 | 0.044 |
|  | GO:0046914 | transition metal ion binding | 8 | 11.59 | 0.051 |
|  | GO:0016684 | oxidoreductase activity, acting on peroxide as acceptor | 2 | 2.90 | 0.073 |
|  | GO:0004601 | peroxidase activity | 2 | 2.90 | 0.073 |

Table S2B: Enriched Down-regulated Gene Ontology Groups in the experiment of furfural challenge during acidogenesis

| GOTERM_Category | GO number | Term | Count | % | P-value |
| --- | --- | --- | --- | --- | --- |
| Biological Process | GO:0006525 | arginine metabolic process | 5 | 14.29 | 0.000 |
|  | GO:0006526 | arginine biosynthetic process | 4 | 11.43 | 0.000 |
|  | GO:0009064 | glutamine family amino acid metabolic process | 5 | 14.29 | 0.000 |
|  | GO:0009084 | glutamine family amino acid biosynthetic process | 4 | 11.43 | 0.000 |
|  | GO:0009308 | amine metabolic process | 8 | 22.86 | 0.001 |
|  | GO:0006520 | cellular amino acid metabolic process | 7 | 20.00 | 0.002 |
|  | GO:0044106 | cellular amine metabolic process | 7 | 20.00 | 0.002 |
|  | GO:0006519 | cellular amino acid and derivative metabolic process | 7 | 20.00 | 0.003 |
|  | GO:0009401 | phosphoenolpyruvate-dependent sugar phosphotransferase system | 6 | 17.14 | 0.004 |
|  | GO:0008643 | carbohydrate transport | 6 | 17.14 | 0.005 |
|  | GO:0043436 | oxoacid metabolic process | 7 | 20.00 | 0.007 |
|  | GO:0019752 | carboxylic acid metabolic process | 7 | 20.00 | 0.007 |
|  | GO:0042180 | cellular ketone metabolic process | 7 | 20.00 | 0.007 |
|  | GO:0006082 | organic acid metabolic process | 7 | 20.00 | 0.007 |
|  | GO:0006810 | transport | 9 | 25.71 | 0.042 |
|  | GO:0051234 | establishment of localization | 9 | 25.71 | 0.042 |
|  | GO:0008652 | cellular amino acid biosynthetic process | 4 | 11.43 | 0.048 |
|  | GO:0051179 | localization | 9 | 25.71 | 0.051 |
|  | GO:0009309 | amine biosynthetic process | 4 | 11.43 | 0.054 |
|  | GO:0046394 | carboxylic acid biosynthetic process | 4 | 11.43 | 0.078 |
|  | GO:0016053 | organic acid biosynthetic process | 4 | 11.43 | 0.081 |
| Cellular Component | GO:0009348 | ornithine carbamoyltransferase complex | 2 | 5.71 | 0.027 |
|  | GO:0005737 | cytoplasm | 10 | 28.57 | 0.027 |
|  | GO:0044424 | intracellular part | 10 | 28.57 | 0.072 |
| Molecular Function | GO:0016740 | transferase activity | 11 | 31.43 | 0.016 |
|  | GO:0004585 | ornithine carbamoyltransferase activity | 2 | 5.71 | 0.021 |
|  | GO:0016743 | carboxyl- or carbamoyltransferase activity | 2 | 5.71 | 0.035 |

Table S2C: Enriched Up-regulated Gene Ontology Groups in the experiment of furfural challenge during solventogenesis

| GOTERM_Category | GO number | Term | Count | % | P-value |
| --- | --- | --- | --- | --- | --- |
| Biological Process | GO:0015698 | inorganic anion transport | 6 | 2.74 | 0.001 |
|  | GO:0006820 | anion transport | 6 | 2.74 | 0.003 |
|  | GO:0006811 | ion transport | 12 | 5.48 | 0.004 |
|  | GO:0031163 | metallo-sulfur cluster assembly | 4 | 1.83 | 0.004 |
|  | GO:0016226 | iron-sulfur cluster assembly | 4 | 1.83 | 0.004 |
|  | GO:0010035 | response to inorganic substance | 3 | 1.37 | 0.015 |
|  | GO:0044248 | cellular catabolic process | 6 | 2.74 | 0.032 |
|  | GO:0006817 | phosphate transport | 3 | 1.37 | 0.035 |
|  | GO:0008272 | sulfate transport | 3 | 1.37 | 0.035 |
|  | GO:0009166 | nucleotide catabolic process | 3 | 1.37 | 0.035 |
|  | GO:0006164 | purine nucleotide biosynthetic process | 6 | 2.74 | 0.052 |
|  | GO:0006163 | purine nucleotide metabolic process | 6 | 2.74 | 0.062 |
|  | GO:0034655 | nucleobase, nucleoside, nucleotide and nucleic acid catabolic process | 3 | 1.37 | 0.075 |
|  | GO:0044270 | nitrogen compound catabolic process | 3 | 1.37 | 0.075 |
|  | GO:0034656 | nucleobase, nucleoside and nucleotide catabolic process | 3 | 1.37 | 0.075 |
|  | GO:0055086 | nucleobase, nucleoside and nucleotide metabolic process | 11 | 5.02 | 0.083 |
|  | GO:0006139 | nucleobase, nucleoside, nucleotide and nucleic acid metabolic process | 41 | 18.72 | 0.093 |
| Molecular Function | GO:0015103 | inorganic anion transmembrane transporter activity | 6 | 2.74 | 0.001 |
|  | GO:0008509 | anion transmembrane transporter activity | 7 | 3.20 | 0.001 |
|  | GO:0043492 | ATPase activity, coupled to movement of substances | 10 | 4.57 | 0.004 |
|  | GO:0042626 | ATPase activity, coupled to transmembrane movement of substances | 10 | 4.57 | 0.004 |
|  | GO:0016820 | hydrolase activity, acting on acid anhydrides, catalyzing transmembrane movement of substances | 10 | 4.57 | 0.005 |
|  | GO:0016887 | ATPase activity | 19 | 8.68 | 0.006 |
|  | GO:0042623 | ATPase activity, coupled | 12 | 5.48 | 0.007 |
|  | GO:0015399 | primary active transmembrane transporter activity | 10 | 4.57 | 0.007 |
|  | GO:0015405 | P-P-bond-hydrolysis-driven transmembrane transporter activity | 10 | 4.57 | 0.007 |
|  | GO:0042625 | ATPase activity, coupled to transmembrane movement of ions | 6 | 2.74 | 0.012 |
|  | GO:0016728 | oxidoreductase activity, acting on CH or CH2 groups, disulfide as acceptor | 3 | 1.37 | 0.025 |
|  | GO:0017111 | nucleoside-triphosphatase activity | 22 | 10.05 | 0.033 |
|  | GO:0016725 | oxidoreductase activity, acting on CH or CH2 groups | 3 | 1.37 | 0.036 |
|  | GO:0015114 | phosphate transmembrane transporter activity | 3 | 1.37 | 0.036 |
|  | GO:0043225 | anion transmembrane-transporting ATPase activity | 3 | 1.37 | 0.036 |
|  | GO:0005315 | inorganic phosphate transmembrane transporter activity | 3 | 1.37 | 0.036 |
|  | GO:0016462 | pyrophosphatase activity | 22 | 10.05 | 0.039 |
|  | GO:0016818 | hydrolase activity, acting on acid anhydrides, in phosphorus-containing anhydrides | 22 | 10.05 | 0.044 |
|  | GO:0016817 | hydrolase activity, acting on acid anhydrides | 22 | 10.05 | 0.044 |

Table S2D: Enriched Down-regulated Gene Ontology Groups in the experiment of furfural challenge during solventogenesis

| GOTERM_Category | GO number | Term | Count | % | P-value |
| --- | --- | --- | --- | --- | --- |
| Biological Process | GO:0040011 | locomotion | 30 | 6.25 | 0.000 |
|  | GO:0007626 | locomotory behavior | 27 | 5.63 | 0.000 |
|  | GO:0042330 | taxis | 27 | 5.63 | 0.000 |
|  | GO:0007610 | behavior | 27 | 5.63 | 0.000 |
|  | GO:0006935 | chemotaxis | 27 | 5.63 | 0.000 |
|  | GO:0007165 | signal transduction | 41 | 8.54 | 0.000 |
|  | GO:0009605 | response to external stimulus | 28 | 5.83 | 0.000 |
|  | GO:0042221 | response to chemical stimulus | 30 | 6.25 | 0.000 |
|  | GO:0050896 | response to stimulus | 35 | 7.29 | 0.000 |
|  | GO:0018106 | peptidyl-histidine phosphorylation | 18 | 3.75 | 0.001 |
|  | GO:0018202 | peptidyl-histidine modification | 18 | 3.75 | 0.001 |
|  | GO:0018193 | peptidyl-amino acid modification | 18 | 3.75 | 0.001 |
|  | GO:0006468 | protein amino acid phosphorylation | 18 | 3.75 | 0.001 |
|  | GO:0050789 | regulation of biological process | 77 | 16.04 | 0.002 |
|  | GO:0050794 | regulation of cellular process | 76 | 15.83 | 0.002 |
|  | GO:0065007 | biological regulation | 77 | 16.04 | 0.003 |
|  | GO:0000160 | two-component signal transduction system (phosphorelay) | 27 | 5.63 | 0.003 |
|  | GO:0043687 | post-translational protein modification | 18 | 3.75 | 0.003 |
|  | GO:0009401 | phosphoenolpyruvate-dependent sugar phosphotransferase system | 22 | 4.58 | 0.004 |
|  | GO:0008643 | carbohydrate transport | 23 | 4.79 | 0.005 |
|  | GO:0006464 | protein modification process | 18 | 3.75 | 0.006 |
|  | GO:0006928 | cell motion | 7 | 1.46 | 0.006 |
|  | GO:0006796 | phosphate metabolic process | 19 | 3.96 | 0.021 |
|  | GO:0016310 | phosphorylation | 18 | 3.75 | 0.023 |
|  | GO:0006793 | phosphorus metabolic process | 19 | 3.96 | 0.023 |
|  | GO:0043412 | biopolymer modification | 18 | 3.75 | 0.053 |
|  | GO:0051179 | localization | 55 | 11.46 | 0.082 |
| Cellular Component | GO:0016020 | membrane | 98 | 20.42 | 0.000 |
|  | GO:0005576 | extracellular region | 5 | 1.04 | 0.004 |
|  | GO:0016021 | integral to membrane | 60 | 12.50 | 0.028 |
|  | GO:0031224 | intrinsic to membrane | 60 | 12.50 | 0.031 |
|  | GO:0009420 | flagellin-based flagellum filament | 3 | 0.63 | 0.033 |
|  | GO:0019861 | flagellum | 8 | 1.67 | 0.052 |
|  | GO:0042995 | cell projection | 8 | 1.67 | 0.052 |
|  | GO:0044425 | membrane part | 60 | 12.50 | 0.065 |
| Molecular Function | GO:0004871 | signal transducer activity | 56 | 11.67 | 0.000 |
|  | GO:0060089 | molecular transducer activity | 56 | 11.67 | 0.000 |
|  | GO:0000156 | two-component response regulator activity | 19 | 3.96 | 0.002 |
|  | GO:0000155 | two-component sensor activity | 19 | 3.96 | 0.004 |
|  | GO:0004673 | protein histidine kinase activity | 19 | 3.96 | 0.004 |
|  | GO:0016775 | phosphotransferase activity, nitrogenous group as acceptor | 19 | 3.96 | 0.004 |
|  | GO:0004672 | protein kinase activity | 19 | 3.96 | 0.008 |
|  | GO:0016773 | phosphotransferase activity, alcohol group as acceptor | 28 | 5.83 | 0.022 |
|  | GO:0008519 | ammonium transmembrane transporter activity | 3 | 0.63 | 0.022 |
|  | GO:0015101 | organic cation transmembrane transporter activity | 3 | 0.63 | 0.022 |
|  | GO:0016758 | transferase activity, transferring hexosyl groups | 6 | 1.25 | 0.033 |
|  | GO:0030246 | carbohydrate binding | 8 | 1.67 | 0.052 |
|  | GO:0016757 | transferase activity, transferring glycosyl groups | 8 | 1.67 | 0.065 |
|  | GO:0004553 | hydrolase activity, hydrolyzing O-glycosyl compounds | 11 | 2.29 | 0.079 |
