# Supplementary material for: Transcriptional analysis of Clostridium beijerinckii NCIMB 8052 to elucidate role of furfural stress during acetone butanol ethanol fermentation
Source: Biotechnol Biofuels. 2013 May 4;6:66. doi: 10.1186/1754-6834-6-66 (PMC3681630; doi:10.1186/1754-6834-6-66)
Supplement: Additional file 3: Table S3 — Significantly regulated KEGG classifications during furfural challenge experiment. [file 1754-6834-6-66-S3.doc]

Table S3: Significantly regulated KEGG classifications during furfural challenge experiment

| Treatment | Gene regulation | KEGG Pathway ID | Pathway Definition | Count | % | P-Value |
| --- | --- | --- | --- | --- | --- | --- |
| Furfural challenge at acidogenic phase | Up-regulation | cbe00740 | Riboflavin metabolism | 3 | 4.35 | 0.002 |
|  |  | cbe00240 | Pyrimidine metabolism | 4 | 5.80 | 0.023 |
|  | Down-regulation | cbe00330 | Arginine and proline metabolism | 7 | 20.00 | 0.000 |
|  |  | cbe00051 | Fructose and mannose metabolism | 6 | 17.14 | 0.000 |
|  |  | cbe00520 | Amino sugar and nucleotide sugar metabolism | 6 | 17.14 | 0.000 |
|  |  | cbe02060 | Phosphotransferase system (PTS) | 5 | 14.29 | 0.021 |
| Furfural challenge at solventogenic phase | UP-regulation | cbe02010 | ABC transporters | 18 | 8.22 | 0.001 |
|  |  | cbe00230 | Purine metabolism | 9 | 4.11 | 0.008 |
|  |  | cbe00040 | Pentose and glucuronate interconversions | 6 | 2.74 | 0.017 |
|  |  | cbe00440 | Phosphonate and phosphinate metabolism | 3 | 1.37 | 0.024 |
|  | Down-regulation | cbe02030 | Bacterial chemotaxis | 20 | 4.17 | 0.000 |
|  |  | cbe02020 | Two-component system | 18 | 3.75 | 0.000 |
|  |  | cbe02060 | Phosphotransferase system (PTS) | 20 | 4.17 | 0.000 |
|  |  | cbe00520 | Amino sugar and nucleotide sugar metabolism | 12 | 2.50 | 0.008 |
|  |  | cbe02040 | Flagellar assembly | 8 | 1.67 | 0.018 |
|  |  | cbe00051 | Fructose and mannose metabolism | 10 | 2.08 | 0.035 |
