# Supplementary material for: Transcriptional analysis of Clostridium beijerinckii NCIMB 8052 to elucidate role of furfural stress during acetone butanol ethanol fermentation
Source: Biotechnol Biofuels. 2013 May 4;6:66. doi: 10.1186/1754-6834-6-66 (PMC3681630; doi:10.1186/1754-6834-6-66)
Supplement: Additional file 4: Table S1 — List of 30 genes and sequences of primers used in validation of microarray analysis by Q-RT-PCR. [file 1754-6834-6-66-S4.doc]

Table S1. List of 30 genes and sequences of primers used in validation of microarray analysis by Q-RT-PCR

| Gene symbol | Gene name | Fold change | | Forward primer sequence (5’-3’) | Reverse primer sequence (5’-3’) |
| --- | --- | --- | --- | --- | --- |
|  |  | Microarray analysis | Q-RT-PCR |  |  |
| Cbei_ 3973 | MerR family transcriptional regulator | 5.43^a^ | 2.86^a^ | AGAACACGAATATGCCTATTGAG | TGAACATTTTCCCTGTGCTTTA |
| Cbei_ 3693 | cobalt ABC transporter ATPase | 7.26^a^ | 2.39^a^ | GCCATTGCTTTAATAGGTGTAA | CTTTGAGAACTCTTCATTTTGC |
| Cbei_ 4519 | acetylglutamate kinase | -5.17^a^ | -1.69^a^ | GTTGTTGGTAGCGTAGCC | TGGAACGTCAGTAAGCAGT |
| Cbei_ 1849 | assembly protein SufB | 4.06 ^a^ | 3.38^a^ | AATGGGTATCAGGTTCTTTTGG | AAGTGTTTGGTGCAGCGTGT |
| Cbei_ 1138 | hypothetical protein | 4.47 ^a^ | 4.70 ^a^ | AACAGGAGCCATTTTAGCAA | GTTGTAAATCCCCTACCTTGAC |
| Cbei_ 0597 | glyceraldehyde-3-phosphate dehydrogenase,  type I | -1.05^a^,  -1.48 | 1.35^a^, 1.25 | GGTGCTCAAAGAGTTCCA | GCTTTCATAGCAGCGTTA |
| Cbei_ 1464 | alcohol dehydrogenase | 3.78^a^ | 4.16^a^ | CTAAAAGAGCAGGGGCAGAT | AAACGCCACGTCAACTCC |
| Cbei_ 4218 | type II secretion system protein E | 4.41^a^ | 1.95^a^ | GCAGCTATAACAGGACATTT | CGCAATAACTCCCACAAC |
| Cbei_  2057 | hypothetical protein | 80.04 | 873.10 | TGCAGTAGCGATTGAACA | TAATCCTGCGGCTAAGAA |
| Cbei_  0329 | groEL chaperonin GroEL | 3.84 | 1.87 | TAGAAGAGCCAGTAAGACAAA | TCCACTATTCCACCTTTTATC |
| Cbei_  0315 | pyruvate formate-lyase | 6.11 | 55.72 | CATATTGGGATGGACACTCA | GCCTAAAGCAAGCCACTC |
| Cbei_  3630 | acetyl-CoA acetyltransferase | 3.04 | 1.77 | GGTGATGCTGATGCTATCG | TTCTCCATGTCCCATTCTTT |
| Cbei_  1435 | heavy metal transport/detoxification protein | 4.26 | 1.32 | CCTAAAGACGGGGACATA | ACAGCAACTCATACCACTACTA |
| Cbei_  4532 | PTS system, N-acetylglucosamine-specific IIBC subunit | -17.89 | 0.34 | TAAAGGGTGTTGGAGGAA | CCAGATACACCTGCTGATTT |
| Cbei_  0685 | alcohol dehydrogenase | -5.74 | -16.68 | CTTTATGTTGGGGTTTGC | CCAATAATCCTACCCTCTTC |
| Cbei_  0554 | carbon starvation protein CstA | -12.64 | -4.89 | CTATCTGGGTTCCATTCAC | GCCATTAGGGAGACAAAA |
| Cbei_  4041 | 2-oxoglutarate ferredoxin oxido-reductase subunit beta | -3.69 | -1.20 | GAGAAGGGGATTCTTATGG | TCCGATGTCAGTTGTAGGT |
| Cbei_  1982 | aldehyde oxidase and xanthine dehydrogenase molybdopterin-binding subunit | -3.23 | -1.26 | CAGGACTGGGATGTAATGAT | GTCGGACAAGTAATGGGT |
| Cbei_  1849 | FeS assembly protein SufB | 24.45 | 114.56 | AATGGGTATCAGGTTCTTTTGG | AAGTGTTTGGTGCAGCGTGT |
| Cbei_  2445 | hypothetical protein | 14.03^a^ , 18.03 | 5.39^a^, 19.56 | ACAGGCGTTATTTTAGCGAG | CCTTGATGTTGAACTGCTGAC |
| Cbei_  2681 | thioredoxin reductase | 3.18^a^ , 9.82 | 2.37^a^, 53.82 | GGTAGAGCCGGATTAGATGC | AACCTGTTATGCTGCGAAAA |
| Cbei_  3616 | XRE family transcriptional regulator | 20.39^a^ , 6.69 | 3.67^a^, 3.43 | AGACCAAATTCGCATGATTTAGA | CATCTTGCCACAAACCTTCTTC |
| Cbei_  3974 | aldo/keto reductase | 9.06^a^ , 3.87 | 2.33^a^, 2.45 | CCATTAGCCCAAGGGACATTA | CGCCATTTGAGCAAGAGTTT |
| Cbei_  2725 | response regulator receiver sensor signal transduction histidine kinase | -4.66^a^ ,  -17.21 | 1.12^a^, 0.81 | AACCTGGCGAAGGAACTG | GGCTGAACCTTCCCCATA |
| Cbei_  3278 | coenzyme A transferase | -3.41^a^ ,  -4.41 | 1.68 | TGGAGCATCAATAAACCC | TTCCCTGCTTGTCTACTTCT |
| Cbei_  4273 | MotA/TolQ/ExbB proton channel | -3.81 ^a^ ,  -4.76 | -3.76^a^, -1.16 | GAGGGTTACAAATGGTGG | CTATAATCTTCATGCCTTGC |
| Cbei_  4911 | PTS system mannose/fructose/sorbose family transporter subunit IID | -3.71^a^ ,  -3.89 | -1.14^a^, 2.28 | CTGGGGAACACTAAGACCT | CCATAGAACATTCCATACCA |
| Cbei_  2421 | iron-containing alcohol dehydrogenase | 2.73^a^ , 1.26 | 1.54^a^, 2.16 | AGTTATTGCGGCAGGAGT | GAACCAGTCGCTGATAGTGT |
| Cbei_  2676 | aldo/keto reductase | -1.44^a^ ,  -1.62 | -1.04^a^, 2.10 | AGAGGATTAAAGGCTGCTAA | GCCCATTCTGCTGGAGTA |
| Cbei_  3904 | Short-chain dehydrogenase/  reductase | 4.14^a^, 1.92 | 2.38^a^, 1.68 | GCAGCCACAAAAGGAGCAGTT | TTCGGTATTTATTGGACCAGGAG |

^a^Fold change when *C. beijerinckii* 8052 was challenged with furfural at acidogenic phase; values without superscript represent fold change when *C. beijerinckii* 8052 was challenged with furfural at solventogenic phase.
