# Supplementary material for: Transcriptional analysis of Clostridium beijerinckii NCIMB 8052 to elucidate role of furfural stress during acetone butanol ethanol fermentation
Source: Biotechnol Biofuels. 2013 May 4;6:66. doi: 10.1186/1754-6834-6-66 (PMC3681630; doi:10.1186/1754-6834-6-66)
Supplement: Additional file 5: Table S5 — Fold change of solvent production genes according to microarray analysis. [file 1754-6834-6-66-S5.doc]

Table S5. Fold change of solvent production genes according to microarray analysis

| Cbei Symbol | Protein/Enzyme | Fold Change (Acidogenesis) | Fold Change (Solventogenesis) |
| --- | --- | --- | --- |
| Cbei_3832 | aldehyde dehydrogenase | 1.02 | -1.61 |
| Cbei_3833 | 3-oxoacid CoA-transferase, A subunit | 1.07 | -1.55 |
| Cbei_3834 | 3-oxoacid CoA-transferase, B subunit | 1.09 | -1.03 |
| Cbei_3835 | Acetoacetate decarboxylase | 1.17 | -1.44 |
| Cbei_0305 | iron-containing alcohol dehydrogenase | -2.14 | -1.08 |
| Cbei_0204 | Butyrate kinase | 1.04 | -1.16 |
| Cbei_4609 | Butyrate kinase | -1.08 | -1.54 |
| Cbei_4006 | Butyrate kinase | 1.02 | -1.18 |
| Cbei_1165 | acetate kinase | 1.42 | 2.52 |
| Cbei_1164 | phosphate acetyltransferase | 1.38 | 2.16 |
| Cbei_0203 | Phosphate butyryltransferase | -1.03 | -1.31 |
| Cbei_2421 | iron-containing alcohol dehydrogenase | 2.73 | 1.26 |
| Cbei_1932 | iron-containing alcohol dehydrogenase | 1.96 | -1.67 |
| Cbei_1722 | iron-containing alcohol dehydrogenase | -1.28 | -1.94 |
| Cbei_2181 | iron-containing alcohol dehydrogenase | 2.38 | -1.26 |
| Cbei_3630 | acetyl-CoA acetyltransferase | 1.61 | 3.04 |
| Cbei_0411 | acetyl-CoA acetyltransferase | -1.701 | -2.88 |
| Cbei_0325 | 3-hydroxybutyryl-CoA dehydrogenase | -1.51 | -1.39 |
| Cbei_4544 | Enoyl-CoA hydratase/isomerase | 1.00 | 2.33 |
| Cbei_2230 | Enoyl-CoA hydratase/isomerase | 1.54 | 2.90 |
| Cbei_2231 | Enoyl-CoA hydratase/isomerase | -1.33 | -1.37 |
| Cbei_2883 | acyl-CoA dehydrogenase domain protein | -1.30 | -1.56 |
| Cbei_2035 | acyl-CoA dehydrogenase domain protein | -1.66 | -2.33 |
| Cbei_0322 | acyl-CoA dehydrogenase domain protein | -1.80 | -1.92 |
| Cbei_4318 | pyruvate flavodoxin/ferredoxin oxidoreductase | -1.39 | -1.16 |
| Cbei_1853 | pyruvate flavodoxin/ferredoxin oxidoreductase | 2.08 | 3.57 |
| Cbei_1458 | pyruvate flavodoxin/ferredoxin oxidoreductase | -1.37 | -3.56 |
| Cbei_4042 | pyruvate flavodoxin/ferredoxin oxidoreductase | 1.11 | -1.85 |
| Cbei_4110 | hydrogenase, Fe-only | -1.20 | 2.21 |
| Cbei_3796 | hydrogenase, Fe-only | -1.24 | 1.00 |
| Cbei_1773 | hydrogenase, Fe-only | 1.40 | 1.10 |
| Cbei_4000 | Ferredoxin hydrogenase | -1.15 | 1.29 |
| Cbei_3006 | hydrogenase expression/formation protein HypE | -3.25 | -1.14 |
| Cbei_3007 | hydrogenase formation HypD protein | -1.42 | -3.00 |
| Cbei_1901 | hydrogenase large subunit domain protein | 1.02 | -1.32 |
| Cbei_0327 | Ferredoxin hydrogenase | -2.06 | -2.73 |
| Cbei_3008 | hydrogenase assembly chaperone hypC/hupF | -1.62 | -3.64 |
| Cbei_3009 | (NiFe) hydrogenase maturation protein HypF | -1.73 | -1.43 |
| Cbei_3011 | hydrogenase maturation protease | -1.67 | -1.78 |
| Cbei_3012 | nickel-dependent hydrogenase, large subunit | -1.32 | -2.70 |
| Cbei_3013 | hydrogenase (NiFe) small subunit HydA | -1.28 | -1.98 |
| Cbei_3696 | hydrogenase accessory protein HypB | 1.39 | -1.07 |
